# Novel miRNA signature for predicting the stage of hepatocellular carcinoma

**DOI:** 10.1038/s41598-020-71324-z

**Published:** 2020-09-02

**Authors:** Srinivasulu Yerukala Sathipati, Shinn-Ying Ho

**Affiliations:** 1 Institute of Bioinformatics and Systems Biology, National Chiao Tung University, Hsinchu, Taiwan; 2 Institute of Population Health Sciences, National Health Research Institutes, Miaoli, Taiwan; 3 Department of Biological Science and Technology, National Chiao Tung University, Hsinchu, Taiwan; 4 Center For Intelligent Drug Systems and Smart Bio-devices (IDS2B), National Chiao Tung University, Hsinchu, Taiwan

**Keywords:** Computational biology and bioinformatics, Machine learning, Hepatocellular carcinoma

## Abstract

Hepatocellular carcinoma (HCC) is one of the leading causes of cancer deaths worldwide. Recently, microRNAs (miRNAs) are reported to be altered and act as potential biomarkers in various cancers. However, miRNA biomarkers for predicting the stage of HCC are limitedly discovered. Hence, we sought to identify a novel miRNA signature associated with cancer stage in HCC. We proposed a support vector machine (SVM)-based cancer stage prediction method, SVM-HCC, which uses an inheritable bi-objective combinatorial genetic algorithm for selecting a minimal set of miRNA biomarkers while maximizing the accuracy of predicting the early and advanced stages of HCC. SVM-HCC identified a 23-miRNA signature that is associated with cancer stages in patients with HCC and achieved a 10-fold cross-validation accuracy, sensitivity, specificity, Matthews correlation coefficient, and area under the receiver operating characteristic curve (AUC) of 92.59%, 0.98, 0.74, 0.80, and 0.86, respectively; and test accuracy and test AUC of 74.28% and 0.73, respectively. We prioritized the miRNAs in the signature based on their contributions to predictive performance, and validated the prognostic power of the prioritized miRNAs using Kaplan–Meier survival curves. The results showed that seven miRNAs were significantly associated with prognosis in HCC patients. Correlation analysis of the miRNA signature and its co-expressed miRNAs revealed that hsa-let-7i and its 13 co-expressed miRNAs are significantly involved in the hepatitis B pathway. In clinical practice, a prediction model using the identified 23-miRNA signature could be valuable for early-stage detection, and could also help to develop miRNA-based therapeutic strategies for HCC.

## Introduction

Hepatocellular carcinoma (HCC) is the most common type of liver cancer, and the third leading cause of cancer deaths worldwide^1^. Hepatitis B and C viral infection^2,3^, cirrhosis^4^, heavy alcoholism^5^, hemochromatosis^6^ and alpha-1-antitrypsin deficiency^7^ are risk factors associated with HCC. Treatment conditions depend on cancer stage, availability of treatment resources, liver function, and clinical expertise^1^. Despite advances in treatment conditions, the overall survival rate of patients with HCC has not improved^8^, largely because most cases of HCC are diagnosed at an advanced stage^9^. Early-stage detection of HCC creates opportunities to use a wider range of treatment options^10^. Hence, early-stage detection of HCC plays a critical role in guiding treatment decisions. Identification of biomarkers for early-stage detection will help to improve treatment strategies and ensure that more patients receive the proper treatment.

Recently, microRNAs (miRNAs) have attracted interest as biomarkers due to their critical roles in cancer development and prognosis. MiRNA dysregulation is observed in multiple types of cancers: in lung cancer, hsa-let-7a expression is associated with poor survival^11^. MiRNAs have been used as biomarkers in HCC^12^. Hsa-miR-155 is highly expressed in breast and colon cancers^13^. Differential expression of miRNAs has been observed in ovarian cancer^14^. In gastric cancer, a seven-miRNA signature has been used to predict overall survival and relapse-free survival^15^.

Several studies have reported dysregulation of miRNAs in HCC. For example, miR-222 deregulation is observed in HCC cell lines^16^. In humans, miR-224 targets glycine N-methyl transferase and plays an important role in HCC tumorigenesis^17^. A 20-miRNA signature is associated with survival in HCC patients^18^. Moreover, some miRNAs are thought to have therapeutic potential in HCC^19^. Previously, Toffannin et al., used miRNA profiling of 89 HCC patients, followed by unsupervised hierarchical clustering, to categorize HCC into three sub classes^20^. Machine learning models have been used to predict the treatment response of trans-arterial chemoembolization in patients with HCC^21^. The random forest method and multiple urine DNA biomarkers have been used for HCC screening^22^. A. Nagy et al. identified 223 miRNAs as prognostic biomarkers based on previous literature and validated their prognostic power using the independent datasets; in which, 55 individual miRNAs are significantly associated with the overall survival of HCC^23^. Previously developed methods and studies have mainly focused on identifying differentially expressed genes and survival variants in HCC. Early stage detection and diagnosis of cancer remains a challenge for clinicians. MiRNAs are considered as potential tumor markers due to their tissue specificity and capability to predict clinicopathological parameters^24^. Several studies have been demonstrated that miRNAs have the potential to be new biomarkers in various cancers for early detection^25–28^. Moreover, miRNAs can be detectable not only from tissue samples but also from a wide range of biological samples, such as urine, blood plasma, and serum. However, few studies have attempted to predict the stage of HCC using the genomic profiling. Therefore, this study aims to identify a miRNA signature consisting of a small set of miRNA biomarkers that can predict the cancer stage of patients with HCC, so that this miRNA signature can be useful for developing gene-based target therapies in HCC.

In this study, we proposed a method for predicting the early and advanced stages of HCC using miRNA expression profiles. We retrieved 348 expression profiles of 540 miRNAs (348*540) from 348 HCC patients from The Cancer Genome Atlas (TCGA) database. Our dataset includes 258 patients with early-stage disease and 90 patients with advanced HCC. We utilized a support vector machine (SVM)-based classifier^29^, SVM-HCC, which incorporated with an inheritable bi-objective combinatorial genetic algorithm (IBCGA)^30^ to identify a miRNA signature capable of distinguishing early-stage patients from advanced-stage HCC. Though optimization technique of the SVM-HCC was adopted from our previous study^31^, identified miRNA signature is novel in HCC stage prediction. The main purpose of this study is to identify a miRNA signature associated with cancer stage of patients with HCC. We ranked the miRNAs in the signature based on their contributions to predictive performance, and subjected the 10 top-ranked miRNAs to further analysis. Next, to investigate the prognostic power of the identified miRNA signature among the patients with HCC, Kaplan–Meier (KM) survival analysis was performed. The expression difference of the 10 top-ranked miRNAs was compared between cancer and normal samples. The biological significance of the identified miRNA signature was analyzed using Kyoto Encyclopedia of Genes and Genomes (KEGG) pathway and Gene Ontology (GO) annotations. Finally, we identified co-expressed miRNAs to the miRNA signature to provide the more information on its overall impact on HCC.

## Results and discussion

**Figure 1:**
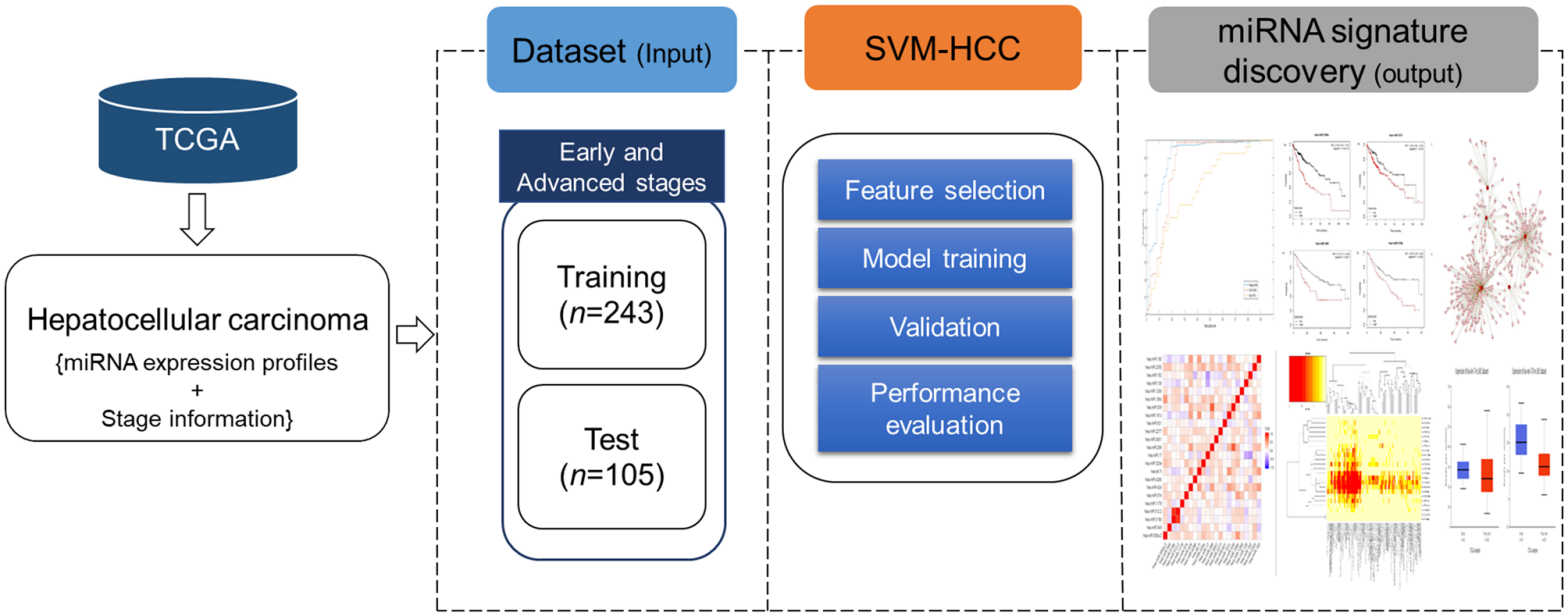
System flowchart representing the dataset, SVM-HCC method and miRNA signature identification.

The proposed method, SVM-HCC, distinguished patients with HCC into early-stage and advanced-stage groups based on their miRNA expression profiles. We used a dataset containing 540 miRNA expression profiles from 348 HCC patients, of whom 248 had early-stage and 90 had advanced-stage HCC. SVM-HCC, used a feature selection algorithm (IBCGA) to select a significant miRNA signature associated with early and advanced stages of HCC. The system flowchart of the overall process is depicted in Fig. 1.

We compared SVM-HCC with standard machine learning methods including sequential minimal optimization (SMO), multilayer perceptron (MLP), naïve Bayes, LibSVM, and random forest. For the feature selection, we used the Ranker search and correlation attribute evaluation method of Waikato Environment for Knowledge Analysis (Weka) to select 30 features to classify early-stage and advanced-stage HCC patients. SVM-HCC performed well relative to these machine learning methods in terms of training accuracy. Using the training set (*n* = 348), SVM-HCC achieved a mean training accuracy, sensitivity, specificity, and Matthews correlation coefficient (MCC) of 89.56 ± 1.27%, 0.94 ± 0.01, 0.73 ± 0.03, and 0.71 ± 0.03, respectively. SVM-HCC achieved the best training accuracy, sensitivity, specificity, MCC, and area under the receiver operating characteristic curve (AUC) of 92.24, 0.96, 0.81, 0.79, and 0.90, respectively. The comparison results are shown in Table 1.

**Table 1:** Comparison of predictive performance of SVM-HCC with those of other machine learning methods.

Method	Training accuracy (%)	Selected miRNAs	Sensitivity	Specificity	MCC	AUC
SMO	72.70	30	0.96	0.05	0.03	0.50
MLP	66.09	30	0.79	0.27	0.07	0.60
Naïve Bayes	73.27	30	0.82	0.46	0.3	0.71
LibSVM	74.71	30	0.99	0.11	0.15	0.54
Random forest	75.28	30	0.94	0.21	0.22	0.72
SVM-HCC	92.24	37	0.96	0.81	0.79	0.90
SVM-HCC-Mean	89.56 ± 1.27	35 ± 3.9	0.94 ± 0.01	0.73 ± 0.03	0.71 ± 0.03	0.86 ± 0.02

Next, to observe the difference in the prediction performance among the standard machine learning methods with the feature size, we used the greedy stepwise search and Cfs Subset Evaluator attribute evaluation method of Weka to select 19 features to classify early and advanced stages of patients with HCC. The prediction performance results shown that only a slight difference in the prediction accuracies was observed for the SMO, MLP, naïve Bayes, LibSVM, and random forest methods when compared to the Table 1. SMO, MLP, naïve Bayes, LibSVM, and random forest methods shown the accuracy differences of 1.15%, 1.43%, 0.86%, 2.87%, and 1.15%, respectively. However, there was no larger difference observed for the AUCs among these methods, shown in Supplementary Table S1.

### The comparison of AUCs

Further, statistical analysis was performed to compare the prediction ability of SVM-HCC with some machine learning methods using the AUC comparison method proposed by Hanley and McNeil^32^. This analysis provides the statistical test comparison between the AUC of SVM-HCC and the AUCs of other machine learning methods. When compared the statistical test AUC of SVM-HCC (AUC = 0.9), SMO obtained a standard error (SE), AUC area difference (AUCd), Z value, and a *p* value of 0.03, 0.40, 10.29, and *p* < 0.001, respectively; MLP obtained SE, AUCd, Z value, and a *p* value of 0.033, 0.3, 8.09, and *p* < 0.001, respectively; naïve Bayes obtained SE, AUCd, Z value, and a *p* value of 0.029, 0.19, 5.71, and *p* < 0.001, respectively; LibSVM obtained SE, AUCd, Z value, and a *p* value of 0.034, 0.36, 9.34, and *p* < 0.001, respectively; and random forest obtained SE, AUCd, Z value, and a *p* value of 0.028, 0.18, 5.48, and *p* < 0.001, respectively. The statistical analysis shows that SVM-HCC method is significantly (*p* < 0.001) different and performed better when compared with the other standard machine learning methods, shown in Supplementary Table S2.

**Figure 2:**
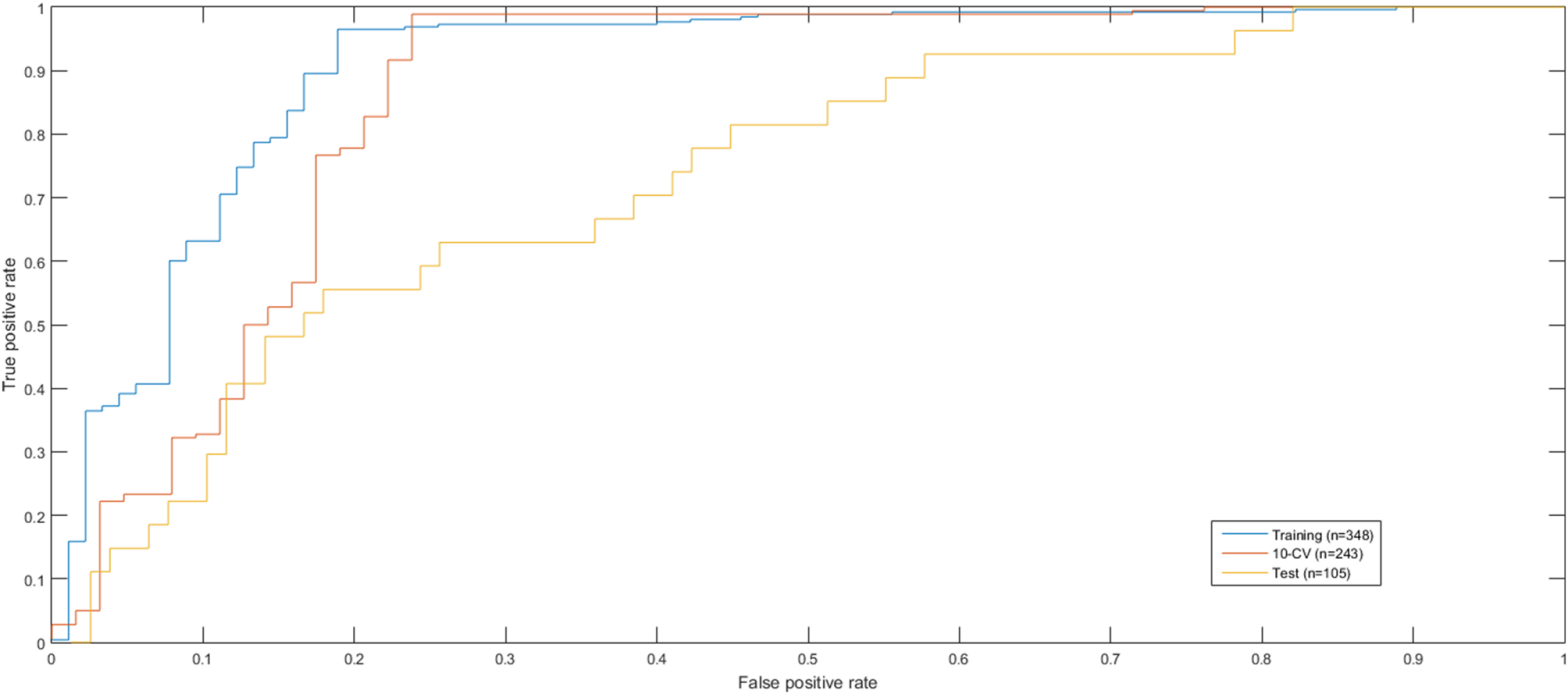
ROC curves for evaluating the predictive performance of SVM-HCC. The ROC curve for Training dataset (AUC = 0.90 using 348-patient HCC cohort), 10-CV (AUC = 0.86 using 243-patient HCC cohort), and test dataset (AUC = 0.73 using 105-patient HCC cohort).

We performed 30 independent runs of SVM-HCC to select a robust miRNA signature using the appearance score^33^, which was calculated based on the frequency of each feature over the independent runs. The most robust miRNA signature had an appearance score of 6.17. SVM-HCC identified a 23-miRNA signature associated with the early and advanced stages of HCC, and achieved a tenfold cross-validation (10-CV) accuracy, sensitivity, specificity, MCC and AUC of 92.59%, 0.98, 0.74, 0.80, and 0.86, respectively, and a test accuracy and test AUC of 74.28% and 0.73, respectively. The predictive performance of SVM-HCC was evaluated using a receiver operating characteristic (ROC) curve, and is shown in Fig. 2. Additionally, to investigate the effect of clinical characteristics on the prediction performance, we added some of the clinical characteristics of patients with HCC such as gender, risk factors, race, hepatitis serology, and vital status to the miRNA signature for the stage prediction. However, addition of these clinical features did not improve the prediction performance of SVM-HCC.

### Ranking of miRNA signature

We ranked the miRNA signature identified by SVM-HCC by main effect difference (MED) analysis^34^. The larger MED score indicates the higher contribution towards the prediction accuracy. In this analysis, miRNAs in the signature were ranked based on their MED scores. The miRNA signature contains 23 miRNAs: in order of decreasing MED scores, hsa-miR-550a, hsa-miR-549, hsa-miR-518b, hsa-miR-512, hsa-miR-1179, hsa-miR-574, hsa-miR-424, hsa-miR-4286, hsa-let-7i, hsa-miR-320a, hsa-miR-17, hsa-miR-299, hsa-miR-3651, hsa-miR-2277, hsa-miR-621, hsa-miR-181c, hsa-miR-539, hsa-miR-106b, hsa-miR-1269, hsa-miR-139, hsa-miR-152, hsa-miR-2355, and hsa-miR-150. Rankings of miRNA signatures and MED scores are provided in Table 2. According to the MED analysis, the 10 top-ranked miRNAs contributed better towards prediction performance when compared to the remaining miRNAs of the signature. Hence, we subjected the 10 top-ranked miRNAs to further analysis.

**Table 2:** Ranking of miRNAs and their corresponding scores, using MED analysis.

Rank	MiRNA	MED score
1	hsa-miR-550a	60.91
2	hsa-miR-549	54.72
3	hsa-miR-518b	51.15
4	hsa-miR-512	50.67
5	hsa-miR-1179	27.73
6	hsa-miR-574	27.02
7	hsa-miR-424	26.62
8	hsa-miR-4286	24.72
9	hsa-let-7i	24.16
10	hsa-miR-320a	22.97
11	hsa-miR-17	22.81
12	hsa-miR-299	22.02
13	hsa-miR-3651	17.02
14	hsa-miR-2277	13.76
15	hsa-miR-621	13.61
16	hsa-miR-181c	13.05
17	hsa-miR-539	12.97
18	hsa-miR-106b	10.83
19	hsa-miR-1269	8.53
20	hsa-miR-139	6.70
21	hsa-miR-152	6.62
22	hsa-miR-2355	3.76
23	hsa-miR-150	2.10

### Validation of top-ranked miRNA prognostics in HCC

**Figure 3:**
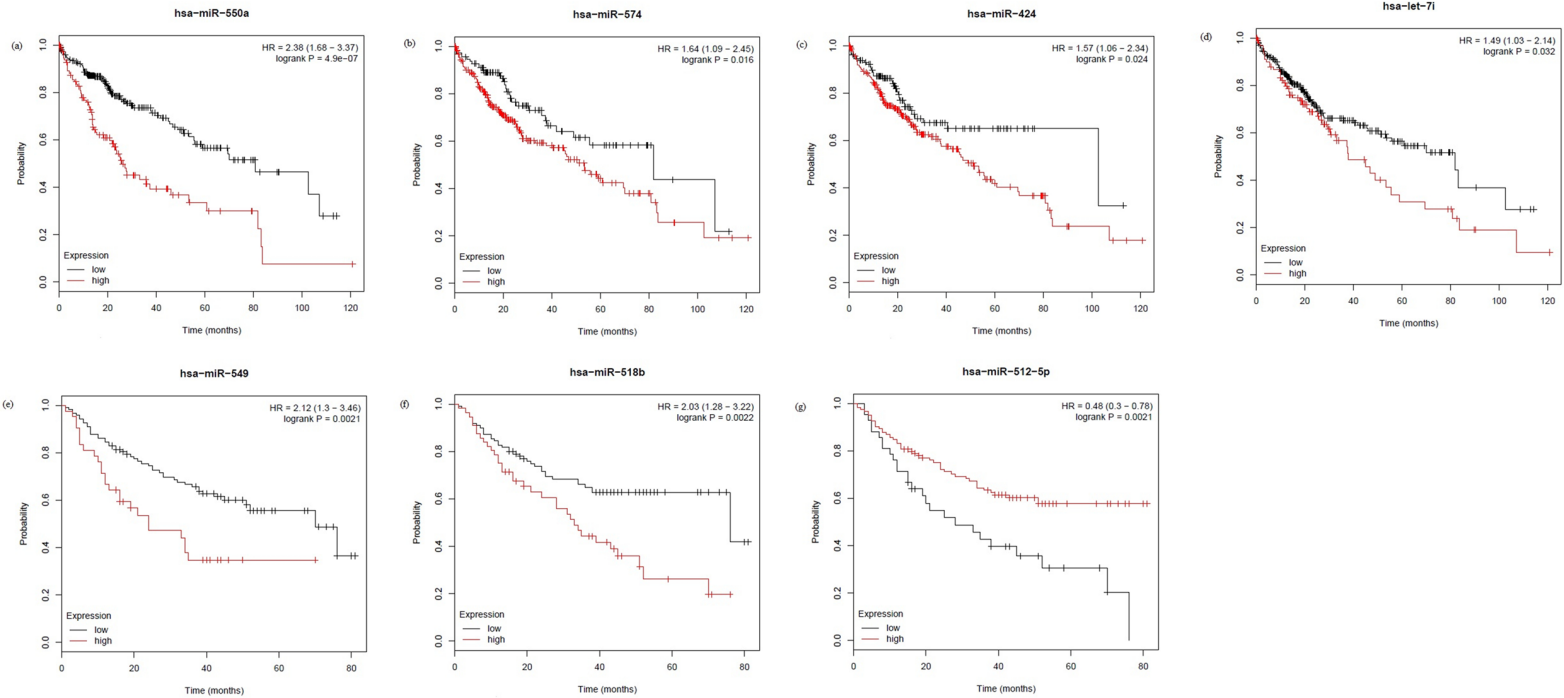
Kaplan–Meier plots of (**a**) hsa-miR-550a, (**b**) hsa-miR-574, (**c**) hsa-miR-424, and (**d**) hsa-let-7i using TCGA dataset, and (**e**) hsa-miR-549, (**f**) hsa-miR-518, and (**g**) hsa-miR-512 using GSE31384 dataset for the high-expression and low-expression groups of the HCC cohort.

We validated the prognostic power of the 10 top-ranked miRNAs in HCC using Kaplan–Meier (KM) survival curves generated by the KM plotter^35^. We selected overall survival data available for 376 patients from TCGA and 166 patients from the GSE31384 dataset, while the median follow-up time of patients in TCGA and GSE31384 was 19.6 and 34 months, respectively. Four of the ten top-ranked miRNAs were significantly associated with overall survival of HCC patients in the TCGA dataset. These four miRNAs, hsa-miR-550a, hsa-miR-574, hsa-miR-424, and hsa-let-7i, had *p* values of 4.9e-07, 0.016, 0.024, and 0.032 and hazard ratios of 2.38, 1.64, 1.57, and 1.49, respectively. Three of the top-ranked miRNAs were significantly associated with overall survival of HCC patients in the GSE31384 dataset, hsa-miR-549, hsa-miR-518, and hsa-miR-512, with *p* values of 0.0021, 0.0022, and 0.0021 and hazard ratios of 2.12, 2.03, and 0.48, respectively. The KM survival curves for the seven miRNAs are shown in Fig. 3. The KM survival curves for the remaining three miRNAs, hsa-miR-1179, hsa-miR-4286, and hsa-miR-320a are shown in Supplementary Fig. S1.

Furthermore, we predicted the prognosis of patients with HCC using remaining 13 miRNAs of the signature across TCGA and GSE31384 datasets. Seven of these 13 miRNAs were significantly associated with the prognosis of patients with HCC across TCGA dataset. Totally, there were 11 of the 23-miRNA signature including, hsa-miR-550a (*p* < 0.001), hsa-miR-574 (*p* = 0.016), hsa-let-7i (*p* = 0.03), hsa-miR-424 (*p* = 0.024), hsa-miR-2277 (*p* = 0.017), hsa-miR-539 (*p* = 0.05), hsa-miR-106b (*p* = 0.011), hsa-miR-1269a (*p* = 0.001), hsa-miR-139 (*p* < 0.001), hsa-miR-2355 (*p* = 0.03), and hsa-miR-150 (*p* = 0.03) were significantly associated with prognosis of patients with HCC across TCGA dataset.

Among the 23-miRNA signature, 10 miRNAs including, hsa-miR-549 (*p* = 0.002), hsa-miR-518b (*p* = 0.002), hsa-miR-512 (*p* = 0.011), hsa-miR-574 (*p* = 0.002), hsa-miR-299 (*p* < 0.001), hsa-miR-181c (*p* = 0.003), hsa-miR-539 (*p* = 0.001), hsa-miR-106b (*p* < 0.001), hsa-miR-152 (*p* < 0.001), and hsa-miR-150 (*p* = 0.031) were significantly associated with prognosis of patients with HCC across GSE31384 dataset.

### Expression difference of top ranked miRNAs in tumor vs normal

We then compared the expression levels of the 10 top-ranked miRNAs in tumor and normal samples using UALCAN web portal^36^, and observed a significant difference in miRNA expression levels between the two groups. Of the top 10 ranked miRNAs, eight miRNAs, hsa-mir-550a, hsa-miR-518b, hsa-miR-512, hsa-miR-574, hsa-miR-424, hsa-miR-4286, hsa-let-7i, hsa-miR-320a are significantly expressed in tumor and normal samples, a *p* value < 0.05 was considered a threshold to describe the statistical significant. Among, two of the ten top-ranked miRNAs (hsa-miR-549 and hsa-miR-1179), the role of hsa-miR-549 was not reported earlier in HCC, and hsa-miR-1179 was not significantly expressed between the tumor and adjacent normal tissues of the TCGA cohort. However, hsa-miR-549 contributed better towards predicting the stage of HCC (MED rank 2) and possessed a significant role in other cancers^37–39^. Though, the expression of hsa-miR-1179 was not significant between the tumor and adjacent normal tissues of the TCGA cohort, a Quantitative Real Time-Polymerase Chain Reaction study on 40 HCC samples reported that hsa-miR-1179 was significantly expressed between HCC and matched normal tissues, and plays an important role in HCC progression and metastasis^40^. The expression levels of the 10 top-ranked miRNAs in the tumor and normal samples are listed in Supplementary Table S3. Box plot representation of relative expression difference of top ranked miRNAs in tumor and normal samples is given in Supplementary Fig. S2. The individual data points of the expression analysis can be accessed from the UALCAN web portal.

Further, we attempted to distinguish the tumor and normal samples using the identified miRNA signature. We used a dataset consisting of 32 normal samples and randomly selected 32 tumor samples, and LibSVM of the WEKA to distinguish the tumor and normal samples. LibSVM achieved a leave-one-out accuracy of 100% to distinguish tumor and normal samples using the 23-miRNA signature.

### Significance of top-ranked miRNAs in cancer

Nine of the ten top-ranked miRNAs are involved in HCC and various other cancers; we summarize their functions in HCC, based on reports in the experimentally validated literature, in Supplementary Table S4.

Hsa-miR-549, the second-ranked miRNA, is differentially expressed in cancer cells relative to normal cells. For example, hsa-miR-549 is highly expressed in colon cancer^37^, colorectal cancer^38^, and breast cancers^39^, with log–fold changes of 0.51, 1.75, and 0.66, respectively, relative to normal cells. However, the role of hsa-miR-549 in HCC has not been reported previously. Our results suggest that hsa-miR-549 is significantly associated with overall survival in HCC patients, and that it actively participates in other major cancers. Hence, it is a worthy subject of further investigation.

Other than the 10 top-ranked miRNAs, several of the remaining miRNAs in the signature are actively involved in HCC and other cancers. For example, expression of hsa-miR-11 is associated with poor prognosis in HCC patients^41^, whereas hsa-miR-299 acts as a tumor suppressor in HCC^42^. Hsa-miR-3651, hsa-miR-621, hsa-miR-181c, hsa-miR-539, hsa-miR-106b, hsa-miR-1269, hsa-miR-139, hsa-miR-152, and hsa-miR-150 are all significantly involved in HCC^43–50^.

We constructed a miRNA target interaction network using Cytoscape^51^ to investigate regulatory interactions compiled in the miRTarBase database. The top-ranked miRNAs annotated with miRBase accession numbers and predicted miRNA interactions using miRTarBase was 2,274. The predicted miRNA target interaction network is shown in Supplementary Fig. S3.

### KEGG pathway and gene ontology enrichment analysis

**Figure 4:**
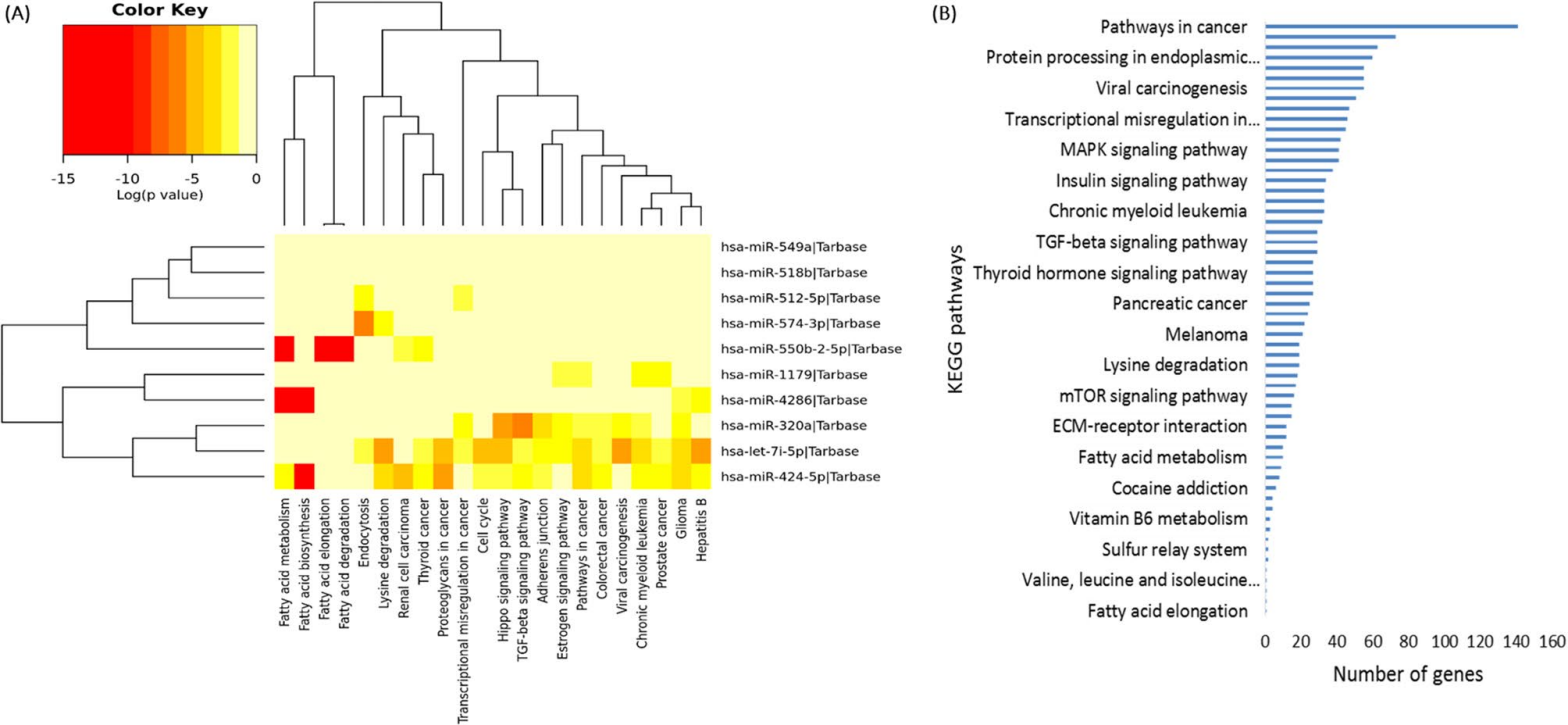
KEGG pathway analysis of the 10 top-ranked miRNAs. (**A**) Heatmap showing enrichment of the 10 top-ranked miRNAs in KEGG pathways. (**B**) The 10 top-ranked miRNAs involved in KEGG pathways are shown in the vertical dimension, and the number of target genes involved in pathways is shown in the horizontal dimension. The size of the pathway is proportional to the number of genes involved in that particular pathway.

Next, we investigated the biological significance of the top-ranked miRNAs using KEGG pathway and GO annotation analysis. First, we used the DIANA-miRPath web tool^52^ to examine their functional annotations. Fisher’s exact test was used for the enrichment analysis. The 10 top-ranked miRNAs are involved in several pathways, the most significant of which are fatty acid metabolism, fatty acid biosynthesis, fatty acid elongation, endocytosis, fatty acid degradation, pathways in cancer, lysine degradation, viral carcinogenesis, glioma, and the Hippo signaling pathway. The top-ranked miRNAs, along with the numbers of predicted target genes in each pathway, are listed in Table 3. The heatmap of the 10 top-ranked miRNAs enriched in KEGG pathways is shown in Fig. 4(A) and the number of target genes involved in pathways is shown in Fig. 4(B). The 23-miRNA signature enriched in KEGG pathways is shown in Supplementary Fig. S4.

**Table 3:** KEGG pathway analysis of the 10 top-ranked miRNAs.

KEGG pathway	Genes	MiRNAs	Adjusted *p* value
Fatty acid metabolism	10	3	< 0.001
Fatty acid biosynthesis	4	2	< 0.001
Fatty acid elongation	1	1	< 0.001
Endocytosis	45	3	< 0.001
Fatty acid degradation	1	1	< 0.001
Pathways in cancer	141	4	< 0.001
Lysine degradation	19	3	< 0.001
Viral carcinogenesis	55	2	< 0.001
Glioma	29	4	< 0.001
Hippo signaling pathway	55	3	< 0.001
Chronic myeloid leukemia	33	4	< 0.001
Hepatitis B	55	3	< 0.001
Proteoglycans in cancer	73	2	< 0.001
TGF-beta signaling pathway	29	3	< 0.001
Adherens junction	33	3	< 0.001
Renal cell carcinoma	24	2	< 0.05
Prostate cancer	41	3	< 0.05
Estrogen signaling pathway	27	3	0.05
Thyroid cancer	12	3	0.07
Cell cycle	51	2	0.06
Transcriptional misregulation in cancer	46	3	0.18
Colorectal cancer	27	3	0.12

Second, we analyzed the involvement of the top-ranked miRNAs in biological pathways, molecular functions, and cellular components using GO annotations. We found that these miRNAs are significantly involved in biological pathways including the mitotic cell cycle, blood coagulation, cellular protein metabolic process, membrane organization, epidermal growth factor receptor signaling pathway, and cell death, with *p* values < 1.11E-16. They are also involved in molecular functions including protein binding transcription factor activity, nucleic acid binding transcription factor activity, ion binding, and RNA binding. Finally, they are involved in cellular components including cytosol, protein complex, neoplasms, and organelles. Details of these associations are given in Supplementary Table S5, and the enrichment of the 23-miRNA signature in GO annotations is shown in Supplementary Fig. S5.

### MiRNAs co-expressed with the top-ranked miRNAs

Identifying more relevant miRNAs may lead to the identification of robust miRNAs that are essential for cancer. Moreover, correlated miRNAs may represent similar biological processes. In its optimization process, SVM-HCC selects a minimal set of biomarkers; hence it selected 23 biomarker miRNAs as a signature associated with HCC stage. However, it might not select some important biomarker miRNAs that are also associated with cancer stage in patients with HCC; also, the priority of miRNA selection may change with the size and number of miRNA profiles used. Hence, to select robust miRNAs outside the 23-miRNA signature, we sought to identify miRNAs that were co-expressed with those 23 miRNAs.

**Figure 5:**
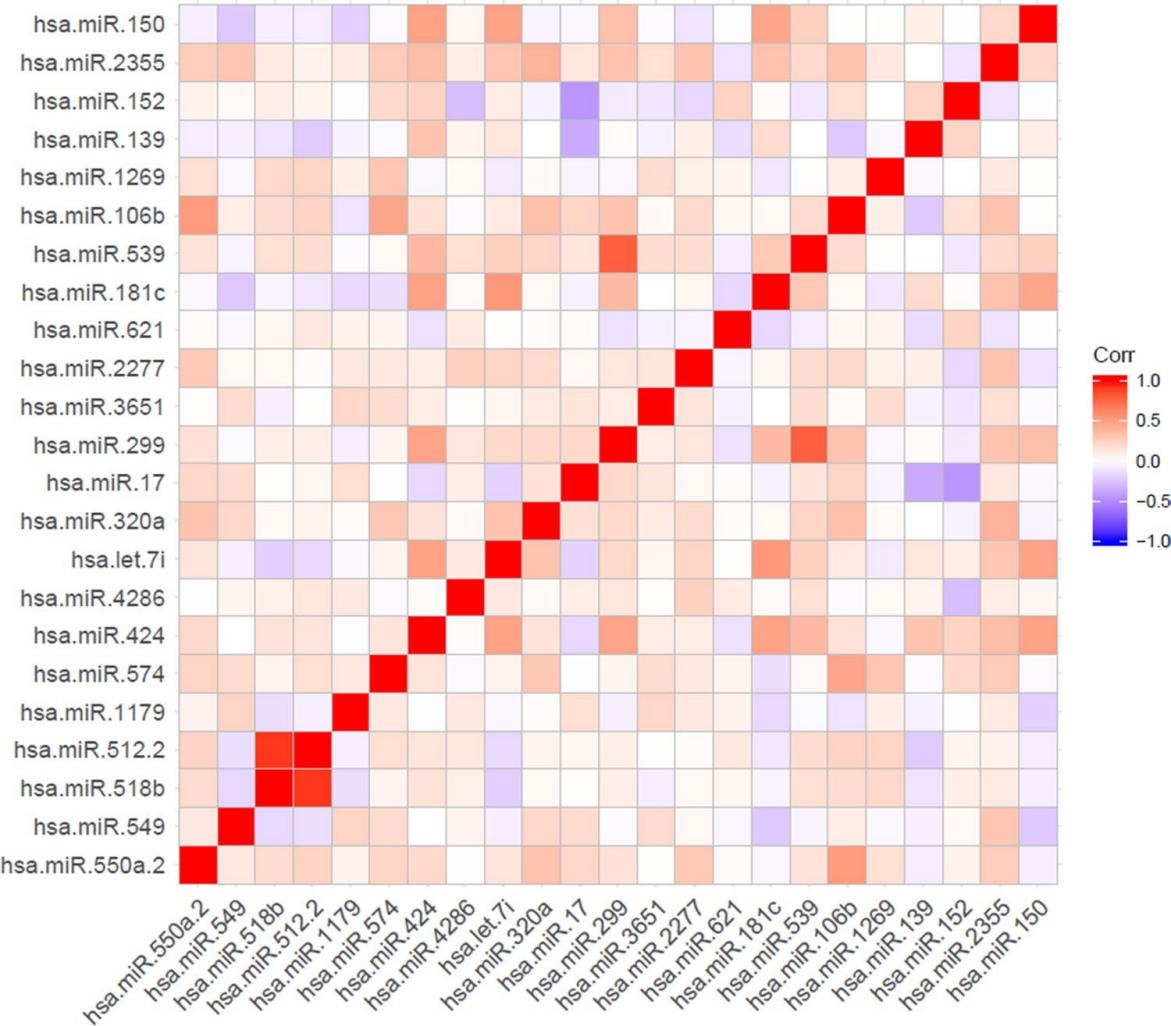
Heatmap showing correlation coefficients among the members of the 23-miRNA signature.

We computed the correlations among each miRNA in the signature using the Pearson correlation coefficient. The miRNAs with the highest correlation coefficient in the signature (0.92) were hsa-miR-512 and hsa-miR-518. These two miRNAs were also significantly associated with overall survival in HCC patients, with *p* values of 0.0022 and 0.0021. Because these miRNAs had high correlation coefficients, we considered them for further analysis. Thus, we sought to analyze the top-ranked individual miRNAs in the signature. The correlation heatmap of the miRNA signature is shown in Fig. 5.

Additionally, we sought to identify the miRNAs that were highly correlated with the miRNA signature in the 540 expression profiles constituting our dataset. To this end, we measured the correlations of the 23-miRNA signature with the 540 miRNA expression profiles. We considered miRNAs with higher correlations to be co-expressed with the signature. These co-expressed miRNAs and their correlation coefficients are listed in Supplementary Table S6. We considered R ≥ 0.5 to be statistically significant. Five miRNAs in the top-ranked miRNA signatures had co-expressed miRNAs with correlations ≥ 0.5. Hsa-miR-518 and hsa-miR-512 had nine co-expressed miRNAs in common. Three miRNAs, hsa-miR-424, hsa-let-7i, and hsa-miR-320a, had 15, 16, and 1 co-expressed miRNA, respectively.

Furthermore, we examined the biological significance of the top-ranked miRNAs and their co-expressed miRNAs to determine whether they were involved in any common pathways. KEGG pathway analysis of hsa-miR-518 and hsa-miR-512 and their co-expressed miRNAs included glycosphingolipid biosynthesis-lacto and neolacto series (hsa00601), folate biosynthesis (hsa00790), one-carbon pool by folate (hsa00670), mucin-type O-Glycan biosynthesis (hsa00512), and central carbon metabolism in cancer (hsa05230). Details of the involvement of hsa-miR-518, hsa-miR-512, and their co-expressed miRNAs in KEGG pathways are provided in Supplementary Table S7. Hsa-miR-424 and its co-expressed miRNAs are significantly involved in several cancer pathways, including proteoglycans in cancer (hsa05205), the Hippo signaling pathway (hsa04390), viral carcinogenesis (hsa05203), pathways in cancer (hsa05200), and glioma (hsa05214). Details of the involvement of hsa-miR-424 and its co-expressed miRNAs in KEGG pathways are provided in Supplementary Table S8. Hsa-let-7i and its co-expressed miRNAs are involved in several cancer pathways, including proteoglycans in cancer, viral carcinogenesis, pathways in cancer, chronic myeloid leukemia, thyroid cancer, bladder cancer, colorectal cancer, glioma, and prostate cancer. Interestingly, we found that hsa-let-7i and its co-expressed miRNAs, hsa-miR-145-5p, hsa-miR-10a-3p, hsa-let-7b-5p, hsa-miR-155-5p, hsa-miR-142-5p, hsa-miR-125a-3p, hsa-miR-199a-5p, hsa-miR-214-3p, hsa-miR-424-3p, hsa-miR-708-3p, hsa-miR-542-5p, hsa-miR-342-5p, and hsa-miR-450a-5p, are significantly (*p* value of 1.18E-10) involved in the hepatitis B pathway (hsa05161), and target 77 genes. Chronic hepatitis B infection has been linked to HCC^53^. Details of the involvement of hsa-let-7i and its co-expressed miRNAs in KEGG pathways are provided in Supplementary Table S9. Experimentally validated gene interactions for hsa-let-7i and its co-expressed miRNAs in the hepatitis B pathway are shown in Supplementary Table S10.

Hsa-miR-320a had a co-expressed miRNA, hsa-miR-1301. These two miRNAs are significantly involved in cancer pathways including transcriptional misregulation in cancer, glioma, viral carcinogenesis, pathways in cancer, colorectal cancer, and pancreatic cancer. Their involvement in biological pathways is shown in detail in Supplementary Table S11. Together, these analyses revealed that not only the 23-miRNA signature, but also its co-expressed miRNAs, are involved in important pathways, and are therefore worthy of further exploration in the context of HCC. These findings could facilitate the development of miRNA-based therapeutic strategies for HCC.

### MiRNAs correlated to the hepatitis infection

We measured the correlation between identified miRNA signature and clinicopathological features of HCC using Spearman correlation coefficient. Three miRNAs, hsa-let-7i, hsa-miR-320a, and hsa-miR-2355 of the signature were significantly correlated with the hepatitis infection, shown in Supplementary Table S12. Additionally, correlation was measured for hsa-let-7i and its 13 co-expressed miRNAs, which were involved in hepatitis B pathway. Two of these miRNAs, hsa-miR-145 and hsa-miR-125a were significantly correlated with the hepatitis infection, shown in Supplementary Table S13.

## Conclusions

Detecting liver cancer at an early stage is difficult because its symptoms often appear only at the later stages. Currently, the diversity of miRNAs, and their differential expression in multiple types of cancer, make them worthy of investigation in the context of cancer research. Recently, miRNAs have been explored as biomarkers of various cancers. Identifying miRNA signatures associated with early-stage HCC could provide useful insight into miRNA-mediated diagnosis of this disease. Developing computational methods for early-stage detection based on miRNA expression could elucidate the variants involved in cancer progression. Besides, potential feature selection methods can easily deal with high-dimensional samples such as gene expression profiles.

In this study, we introduced a SVM-based prediction method, SVM-HCC, which incorporates an optimal feature selection algorithm (IBCGA) to identify miRNA signatures capable of distinguishing early-stage and advanced-stage patients with HCC. SVM-HCC identified a 23-miRNA signature associated with early-stage and advanced-stage HCC, and achieved a 10-CV mean accuracy, sensitivity, specificity, and MCC of 92.44 ± 0.99, 0.96 ± 0.01, 0.78 ± 0.03, and 0.79 ± 0.02, respectively. We prioritized the 23-miRNA signature based on MED scores; miRNAs with higher MED score contributed more to prediction accuracy. The highest-ranked miRNAs were subjected to further analysis.

We validated the prognostic power of the top-ranked miRNAs in HCC using KM survival curves. The results revealed that 7 of the 10 top-ranked miRNAs, hsa-miR-550a, hsa-miR-574, hsa-miR-424, hsa-let-7i, hsa-miR-549, hsa-miR-518, and hsa-miR-512, were significantly associated with overall survival in patients with HCC. In addition, the top-ranked miRNAs are all significantly involved in HCC, with the exception of hsa-miR-549. This miRNA plays an important role in other cancers, but its role in HCC had not been reported previously. However, our results suggest that hsa-miR-549 is significantly associated with overall survival in patients with HCC, and is therefore worthy of further investigation. KEGG pathway and GO enrichment analyses revealed the functional mechanisms of top-ranked miRNAs in several cancer and non-cancer pathways. Although, the identified miRNA signature is potential to predict the stage of HCC, additional information on co-expressed miRNAs to the miRNA signature was provided to explore the possible miRNAs beyond this 23-miRNA signature that might provide specific information/knowledge on its overall impact on HCC. Interestingly, we found that hsa-let-7i and its 13 co-expressed miRNAs were significantly involved in the hepatitis B pathway.

Together, our findings help to explore the role of miRNAs in HCC, and could facilitate early-stage detection and prevention.

## Materials and methods

### Dataset

From the TCGA database, we retrieved a dataset containing miRNA expression profiles from 348 patients with liver HCC; these profiles were obtained using the Illumina HiSeq 2000 platform. After filtering, the final dataset contained 540 miRNA expression profiles from 348 patients. The HCC stage system in the TCGA dataset was based on the size of the primary breast tumor (T), the spread of cancer to lymph nodes (N) and distant metastasis (M) according to the American Joint Committee on Cancer. For classification purposes, the dataset was divided into early-stage (stages 1 and 2) and advanced-stage (stages 3 and 4) groups. There were 258 patients in the early-stage group and 90 patients in the advanced-stage group. Clinical characteristics of the patients with HCC used in this current study is displayed in Supplementary Fig. S6.

### Establishing the SVM-HCC

We proposed a method, SVM-HCC, to identify a miRNA signature capable of distinguishing early-stage and advanced-stage HCC based on miRNA expression profiles. SVM-HCC is based on an SVM^29^ incorporating the feature selection algorithm IBCGA. SVMs are powerful statistical learning algorithms that use non-linear transformation to map data from input space to higher-dimensional space to identify better predictive models. SVMs have become popular in the biomedical sciences, especially in cancer research, due to their potential predictive performance^54^.

We used miRNA expression profiles of patients with HCC as inputs. SVMs work implicitly by only computing the corresponding kernels in the feature space between two data points, *x*_*i*_ and *x*_*j*_. The SVM kernel function is defined as

1 $$K\left( {x_{i} ,x_{j} } \right) = \varphi \left( {x_{i} } \right).\varphi \left( {x_{i} } \right)$$

where $\varphi \left( x \right)$ is the mapping function. SVM-HCC was developed using the LIBSVM package^55^, where the radial basis function (RBF) is used as the kernel function for the implementation of the SVM. RBF is defined as follows:

2 $$K\left( {x_{i} , x_{j} } \right) = {\exp}\left( { - \gamma x_{i} - x_{j} } \right)^{2}$$

In this study, the SVM parameters *C* and γ were optimized based on 10-CV. While establishing the SVM-HCC, an optimal feature selection algorithm, IBCGA, was incorporated into the SVM. IBCGA is an intelligent evolutionary algorithm^56^ that uses an orthogonal array crossover to solve large parameter optimization problems. In the optimization process, IBCGA selects a minimum number of features, in this case miRNAs, while improving its predictive performance. We have successfully applied IBCGA to various types of cancer predictions^31,33,57,58^. To distinguish early-stage from advanced-stage HCC, the parameter settings of SVM and IBCGA were encoded into binary “genes.” In this study, genetic algorithm (GA) terms were used to represent the genes and “chromosomes.” We used 540 miRNA (*m* = 540) expression profiles from 348 HCC patients (*n* = 348) as input. IBCGA parameters were *r*_*start*_ = 10, *r*_*end*_ = 50, N_*pop*_ = 50, and G_*max*_ = 60, as used in^31^. The steps involved in IBCGA are as follows.

Step 1: (Evaluation) Evaluate the fitness value of all individuals using the fitness function, which is the prediction accuracy in terms of 10-CV.

3 $$Accuracy = \frac{TP + TN}{{TP + TN + FP + FN}}$$

Step 2: (Selection) Use a tournament selection method that selects the winner from two randomly selected individuals to generate a mating pool.

Step 3: (Crossover) Select two parents from the mating pool to perform an orthogonal array crossover operation.

Step 4: (Mutation) Apply a conventional mutation operator to randomly selected individuals in the new population. To prevent the highest fitness value from deteriorating, mutation is not applied to the best individuals.

Step 5: (Termination test) If the stopping condition for obtaining the solution is satisfied, then output the best individual as the solution. Otherwise, go to Step 2.

Step 6: (Inheritance) If *r* < *r*_*end*_, randomly change one bit in the binary GA genes for each individual from 0 to 1; increase the number *r* by one, and go to Step 2. Otherwise, stop the algorithm.

### Weka classifier

We used Weka^59^, a powerful data mining tool that uses well-known machine learning algorithms. We compared the predictive performance of SVM-HCC with those of some machine learning methods such as SMO, MLP, naïve Bayes, LIBSVM, and random forest. We performed 10-CV to evaluate the performance of the machine learning models.

### Evaluation metrics

We evaluated the predictive performance of the classifier using the following evaluation metrics: sensitivity (SN), specificity (SP), Matthews correlation coefficient (*MCC*), accuracy (*ACC*), and area under the ROC curve (AUC).

4 $${\text{SN}} = \frac{TP}{{TP + FN}}$$

5 $${\text{SP}} = \frac{TN}{{TN + FP}}$$

6 $${\text{MCC}} = { }\frac{TP \times TN - FP \times FN}{{\sqrt {\left( {TP + FP} \right)\left( {TP + FN} \right)\left( {TN + FP} \right)\left( {TN + FN} \right)} }}$$

7 $$Accuracy = \frac{TP + TN}{{TP + TN + FP + FN}}$$

where TP is true positive, TN is true negative, FP is false positive, and FN is false negative.

## Supplementary information

Supplementary file1

## Data Availability

All data analyzed during this study are publicly available at TCGA data portal (https://portal.gdc.cancer.gov/).

## References

[1] El-Serag HB (2011). Hepatocellular carcinoma. N Engl J Med.

[2] de Martel C (2012). Global burden of cancers attributable to infections in 2008: a review and synthetic analysis. Lancet Oncol.

[3] Beasley RP (1988). Hepatitis B virus. The major etiology of hepatocellular carcinoma. Cancer.

[4] Forner A, Llovet JM, Bruix J (2012). Hepatocellular carcinoma. Lancet.

[5] Lin CW (2013). Heavy alcohol consumption increases the incidence of hepatocellular carcinoma in hepatitis B virus-related cirrhosis. J Hepatol.

[6] Crownover BK, Covey CJ (2013). Hereditary hemochromatosis. Am Fam Physician.

[7] Stoller, J. K., Lacbawan, F. L. & Aboussouan, L. S. in *GeneReviews((R))* (eds M. P. Adam *et al.*) (University of Washington, Seattle University of Washington, Seattle. GeneReviews is a registered trademark of the University of Washington, Seattle. All rights reserved., 1993).

[8] Blum HE (2005). Treatment of hepatocellular carcinoma. Best Pract Res Clin Gastroenterol.

[9] Chen CH (2006). Long-term trends and geographic variations in the survival of patients with hepatocellular carcinoma: analysis of 11,312 patients in Taiwan. J Gastroenterol Hepatol.

[10] Marrero JA (2013). Current treatment approaches in HCC. Clin Adv Hematol Oncol.

[11] Yanaihara N (2006). Unique microRNA molecular profiles in lung cancer diagnosis and prognosis. Cancer Cell.

[12] Mohamed AA (2017). MicroRNAs and clinical implications in hepatocellular carcinoma. World J Hepatol.

[13] Volinia S (2006). A microRNA expression signature of human solid tumors defines cancer gene targets. Proc Natl Acad Sci USA.

[14] Iorio MV (2007). MicroRNA signatures in human ovarian cancer. Can. Res..

[15] Li X (2010). Survival prediction of gastric cancer by a seven-microRNA signature. Gut.

[16] Wong QWL (2008). MicroRNA-223 is commonly repressed in hepatocellular carcinoma and potentiates expression of Stathmin1. Gastroenterology.

[17] Hung J-H (2018). MicroRNA-224 down-regulates Glycine N-methyltransferase gene expression in Hepatocellular Carcinoma. Sci Rep.

[18] Wei R (2013). Clinical significance and prognostic value of microRNA expression signatures in hepatocellular carcinoma. Clin. Cancer Res..

[19] Borel F, Konstantinova P, Jansen PLM (2012). Diagnostic and therapeutic potential of miRNA signatures in patients with hepatocellular carcinoma. J. Hepatol..

[20] Toffanin S (2011). MicroRNA-based classification of hepatocellular carcinoma and oncogenic role of miR-517a. Gastroenterology.

[21] Abajian A (2018). Predicting treatment response to intra-arterial therapies for hepatocellular carcinoma with the use of supervised machine learning—an artificial intelligence concept. J Vasc Interv Radiol.

[22] Wang J (2018). Development and evaluation of novel statistical methods in urine biomarker-based hepatocellular carcinoma screening. Sci Rep.

[23] Nagy Á, Lánczky A, Menyhárt O, Győrffy B (2018). Validation of miRNA prognostic power in hepatocellular carcinoma using expression data of independent datasets. Sci Rep.

[24] Lu J (2005). MicroRNA expression profiles classify human cancers. Nature.

[25] Chen X (2012). Identification of ten serum microRNAs from a genome-wide serum microRNA expression profile as novel noninvasive biomarkers for nonsmall cell lung cancer diagnosis. Int. J. Cancer.

[26] Zhu C (2014). A five-microRNA panel in plasma was identified as potential biomarker for early detection of gastric cancer. Br. J. Cancer.

[27] Wang LG, Gu J (2012). Serum microRNA-29a is a promising novel marker for early detection of colorectal liver metastasis. Cancer Epidemiol..

[28] Kahraman M (2018). MicroRNA in diagnosis and therapy monitoring of early-stage triple-negative breast cancer. Sci Rep.

[29] Vapnik VN (1999). An overview of statistical learning theory. IEEE Trans. Neural Netw.

[30] Shinn-Ying H, Jian-Hung C, Meng-Hsun H (2004). Inheritable genetic algorithm for biobjective 0/1 combinatorial optimization problems and its applications. IEEE Trans Syst. Man Cybern. Part B Cybern..

[31] Yerukala Sathipati S, Ho S-Y (2018). Identifying a miRNA signature for predicting the stage of breast cancer. Sci. Rep..

[32] Hanley JA, McNeil BJ (1982). The meaning and use of the area under a receiver operating characteristic (ROC) curve. Radiology.

[33] Yerukala Sathipati S, Ho S-Y (2017). Identifying the miRNA signature associated with survival time in patients with lung adenocarcinoma using miRNA expression profiles. Sci. Rep..

[34] Tung CW, Ho SY (2008). Computational identification of ubiquitylation sites from protein sequences. BMC Bioinform..

[35] Nagy Á, Lánczky A, Menyhárt O, Győrffy B (2018). Validation of miRNA prognostic power in hepatocellular carcinoma using expression data of independent datasets. Sci. Rep..

[36] Chandrashekar DS (2017). UALCAN: a portal for facilitating tumor subgroup gene expression and survival analyses. Neoplasia (New York, NY).

[37] Sarver AL (2009). Human colon cancer profiles show differential microRNA expression depending on mismatch repair status and are characteristic of undifferentiated proliferative states. BMC Cancer.

[38] Balaguer F (2011). Colorectal cancers with microsatellite instability display unique miRNA profiles. Clin. Cancer Res..

[39] Lee CH (2013). MicroRNA-regulated protein-protein interaction networks and their functions in breast cancer. Int. J. Mol. Sci..

[40] Gao HB, Gao FZ, Chen XF (2019). MiRNA-1179 suppresses the metastasis of hepatocellular carcinoma by interacting with ZEB2. Eur. Rev. Med. Pharmacol. Sci..

[41] Zheng J, Dong P, Gao S, Wang N, Yu F (2013). High expression of serum miR-17-5p associated with poor prognosis in patients with hepatocellular carcinoma. Hepatogastroenterology.

[42] Dang S (2018). MiR-299-3p functions as a tumor suppressor via targeting Sirtuin 5 in hepatocellular carcinoma. Biomed. Pharmacother..

[43] Zhu HR (2018). Microarray expression profiling of microRNAs reveals potential biomarkers for hepatocellular carcinoma. Tohoku J. Exp. Med..

[44] Zhang Y (2018). Downregulated miR-621 promotes cell proliferation via targeting CAPRIN1 in hepatocellular carcinoma. Am. J. Cancer Res..

[45] Ding M (2015). Integrated analysis of miRNA, gene, and pathway regulatory networks in hepatic cancer stem cells. Journal of translational medicine.

[46] Liu Y (2017). miR-539 inhibits FSCN1 expression and suppresses hepatocellular carcinoma migration and invasion. Oncol. Rep..

[47] Jiang L, Li X, Cheng Q, Zhang BH (2015). Plasma microRNA might as a potential biomarker for hepatocellular carcinoma and chronic liver disease screening. Tumour Biol..

[48] Yang XW (2014). MicroRNA-1269 promotes proliferation in human hepatocellular carcinoma via downregulation of FOXO1. BMC Cancer.

[49] Mo Y (2017). Long non-coding RNA XIST promotes cell growth by regulating miR-139-5p/PDK1/AKT axis in hepatocellular carcinoma. Tumour Biol..

[50] Zhou J (2017). MicroRNA-152 inhibits tumor cell growth by directly targeting RTKN in hepatocellular carcinoma. Oncol. Rep..

[51] Shannon P (2003). Cytoscape: a software environment for integrated models of biomolecular interaction networks. Genome Res..

[52] Vlachos IS (2015). DIANA-miRPath v3.0: deciphering microRNA function with experimental support. Nucl. Acids Res..

[53] Di Bisceglie AM (2009). Hepatitis B and hepatocellular carcinoma. Hepatology (Baltimore, MD).

[54] Chu F, Wang L (2005). Applications of support vector machines to cancer classification with microarray data. Int. J. Neural Syst..

[55] Chang C-C, Lin C-J (2011). LIBSVM: A library for support vector machines. ACM Trans. Intell. Syst. Technol..

[56] Shinn-Ying H, Li-Sun S, Jian-Hung C (2004). Intelligent evolutionary algorithms for large parameter optimization problems. IEEE Trans. Evol. Comput..

[57] Yerukala Sathipati S, Huang H-L, Ho S-Y (2016). Estimating survival time of patients with glioblastoma multiforme and characterization of the identified microRNA signatures. BMC Genom..

[58] Yerukala Sathipati S, Sahu D, Huang H-C, Lin Y, Ho S-Y (2019). Identification and characterization of the lncRNA signature associated with overall survival in patients with neuroblastoma. Sci. Rep..

[59] Frank E, Holmes G, Witten IH, Trigg L, Hall M (2004). Data mining in bioinformatics using Weka. Bioinformatics.

