# Supplementary material for: Novel miRNA signature for predicting the stage of hepatocellular carcinoma
Source: Sci Rep. 2020 Sep 2;10:14452. doi: 10.1038/s41598-020-71324-z (PMC7467934; doi:10.1038/s41598-020-71324-z)
Supplement: Supplementary file 1 — Supplementary file1 [file 41598_2020_71324_MOESM1_ESM.docx]

**Novel miRNA signature for predicting the stage of hepatocellular carcinoma**

**Srinivasulu** **Yerukala Sathipati^1,2^ and Shinn-Ying Ho^1, 3,4*^**

^1^Institute of Bioinformatics and Systems Biology, National Chiao Tung University, Hsinchu, Taiwan

^2^Institute of Population Health Sciences, National Health Research Institutes, Miaoli, Taiwan

^3^Department of Biological Science and Technology, National Chiao Tung University, Hsinchu, Taiwan

^4^ Center For Intelligent Drug Systems and Smart Bio-devices (IDS2B), National Chiao Tung University, Hsinchu, Taiwan

*Corresponding author

Correspondence should be addressed to SYH ([syho@mail.nctu.edu.tw](mailto:syho@mail.nctu.edu.tw))

# Supplementary Figures


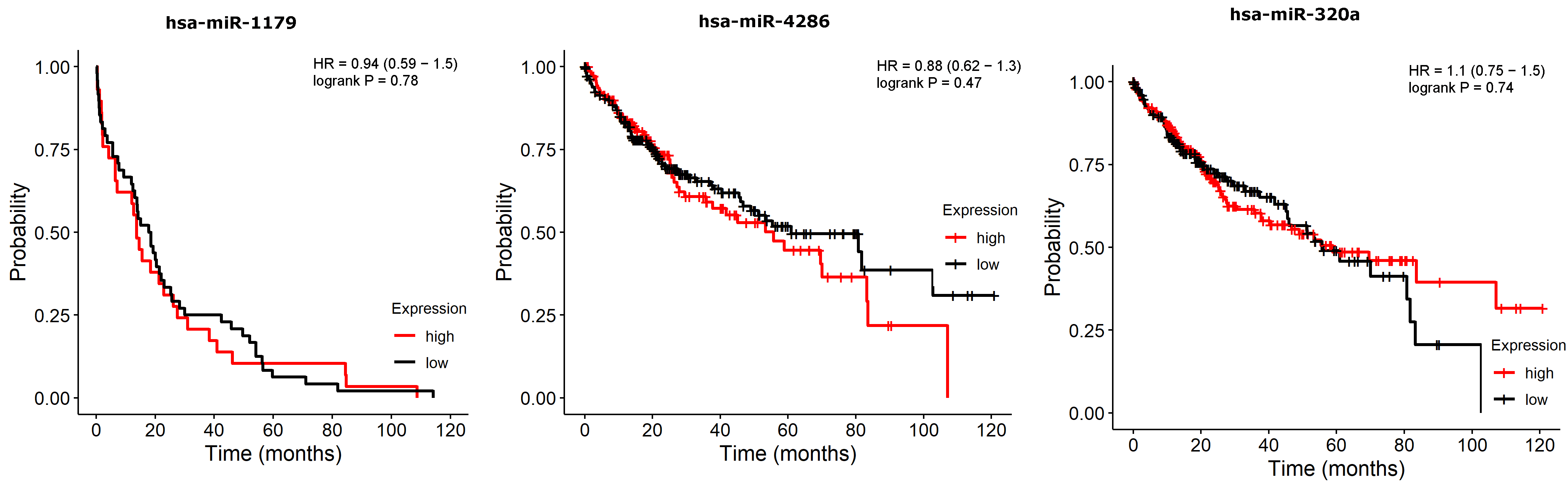


**Supplementary Fig.S1** Kaplan–Meier plots of (a) hsa-miR-1179, (b) hsa-miR-4286, and (c) hsa-miR-320a for the high-expression and low-expression groups of the HCC cohort using the TCGA dataset


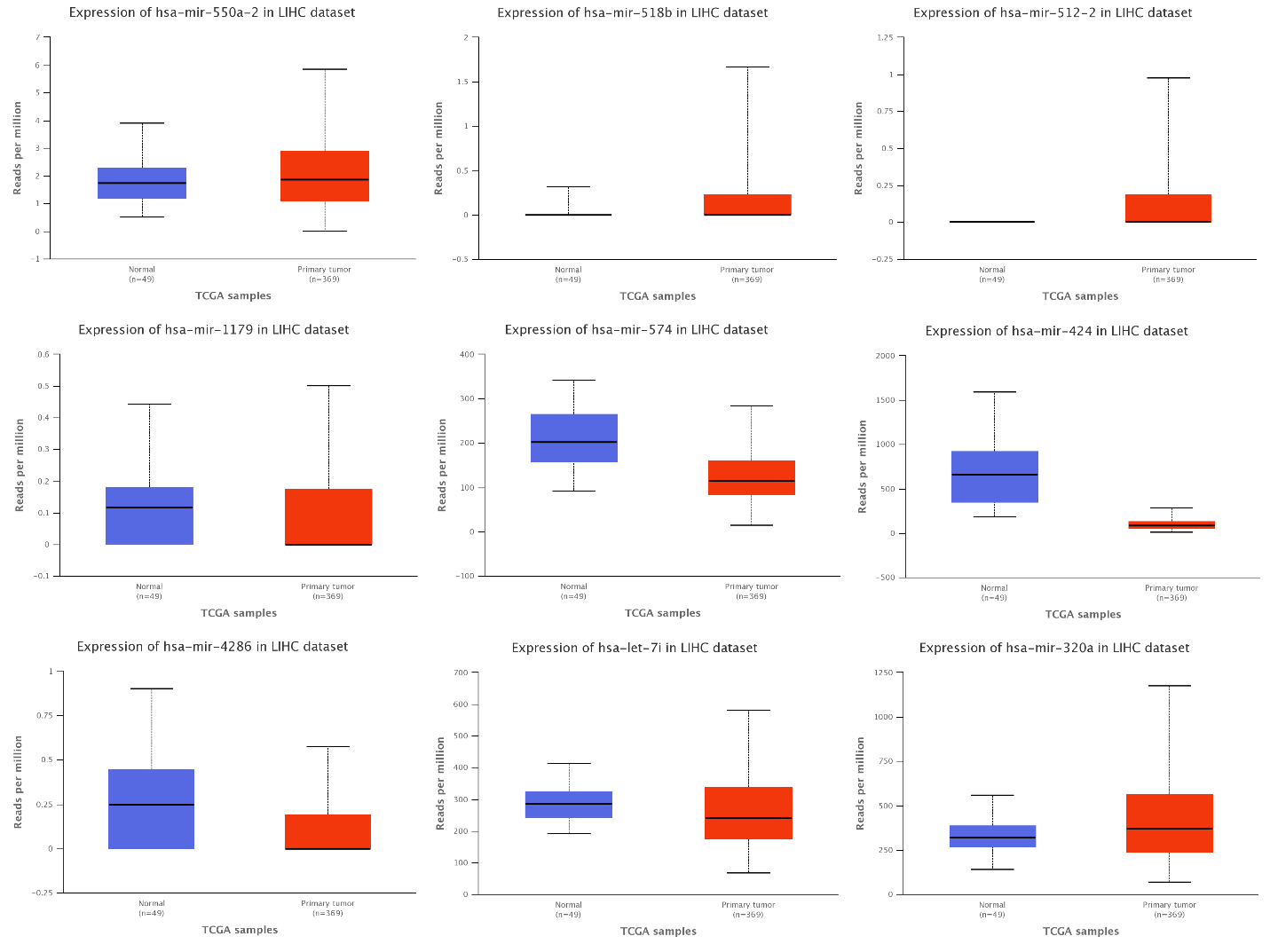


**Supplementary Figure S2**. Expression difference of top 10 ranked miRNAs in tumor and normal samples


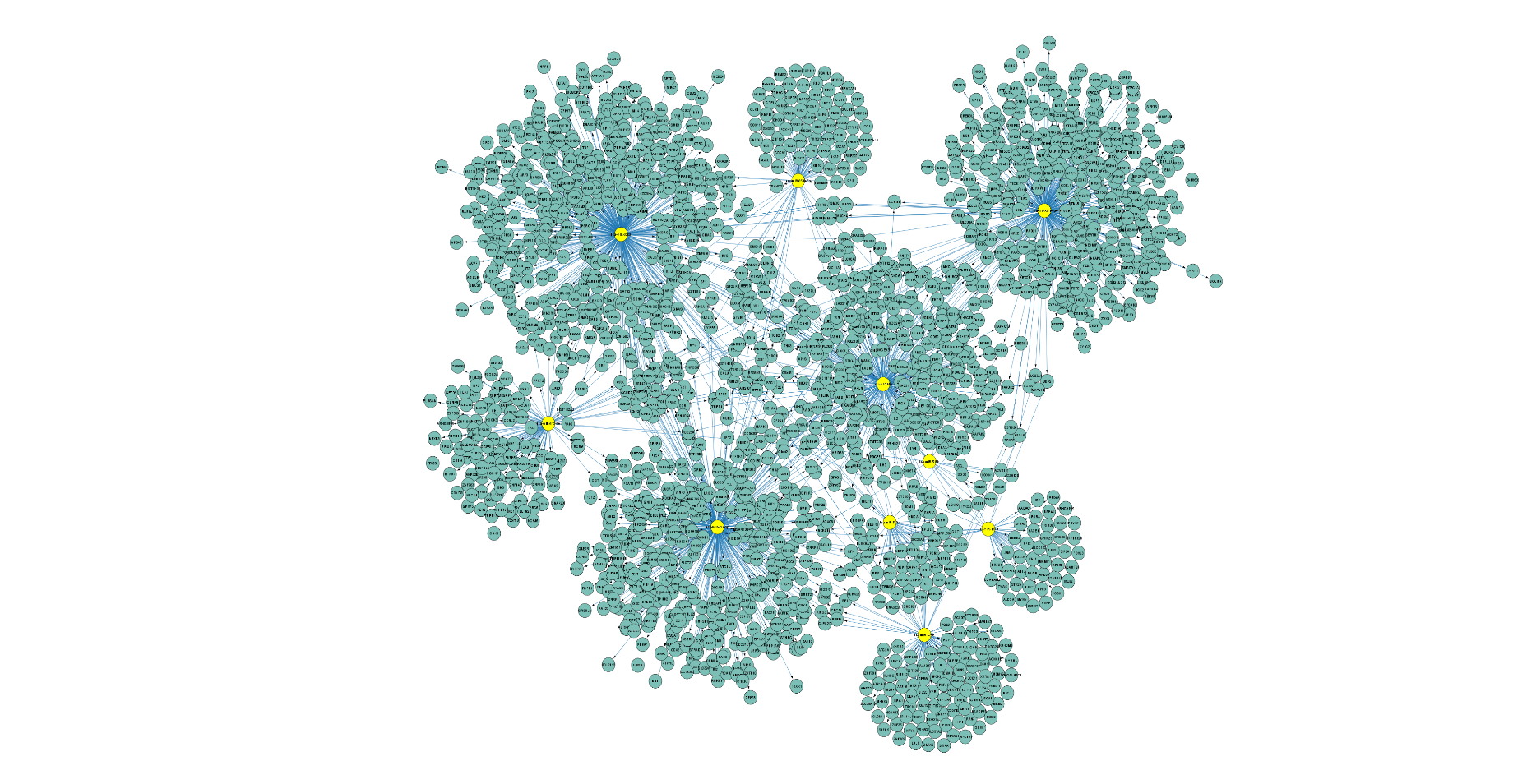


**Supplementary Figure S3**. miRNA and target gene network. The target genes of the 10 top-ranked miRNAs were predicted using the miRTarBase. In this network, microRNAs and target genes are shown as yellow circles and cadet blue circles, respectively, using CyTargetLinker


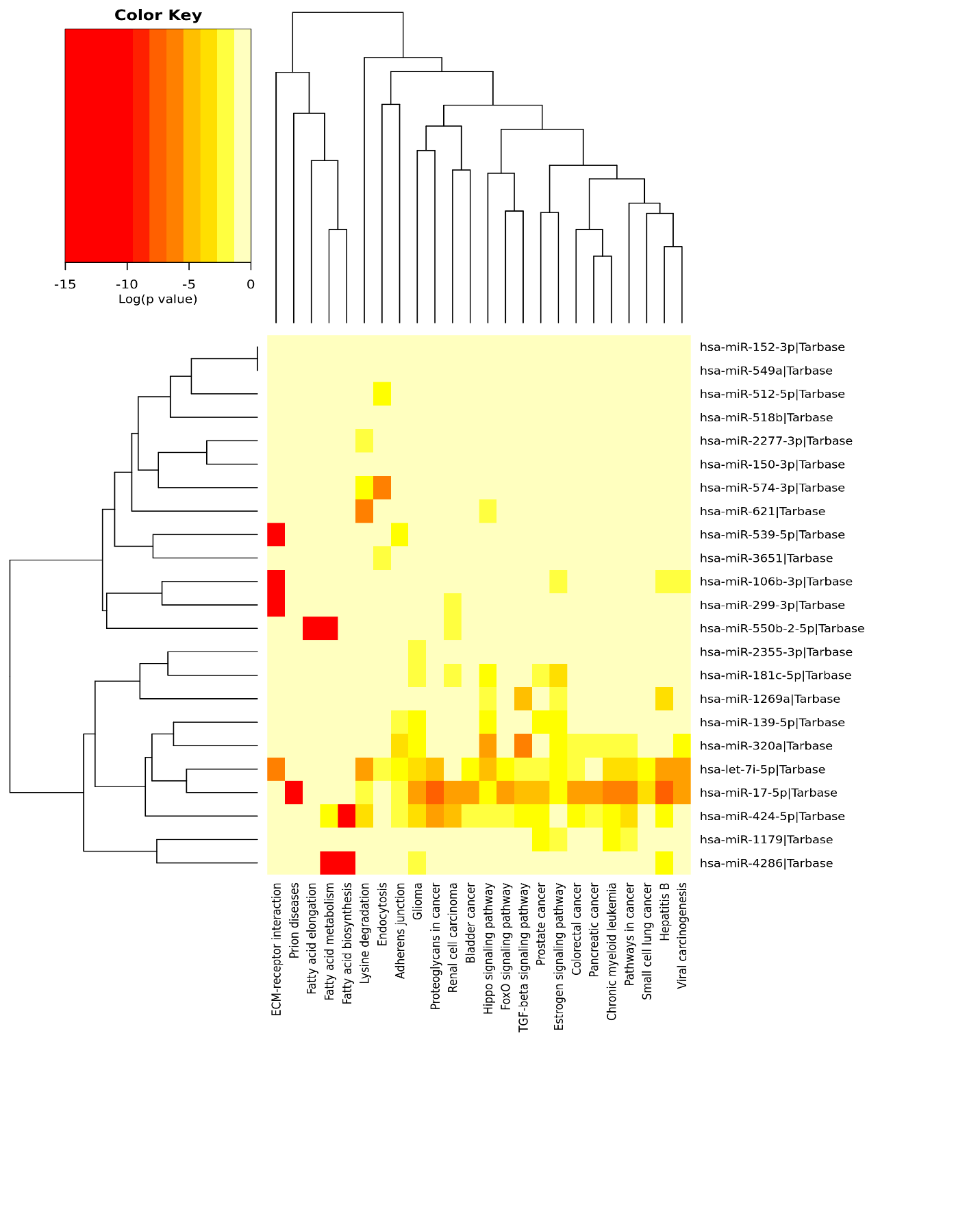


**Supplementary Figure S4**. Heatmap showing enrichment of 23-miRNA signature in KEGG pathways


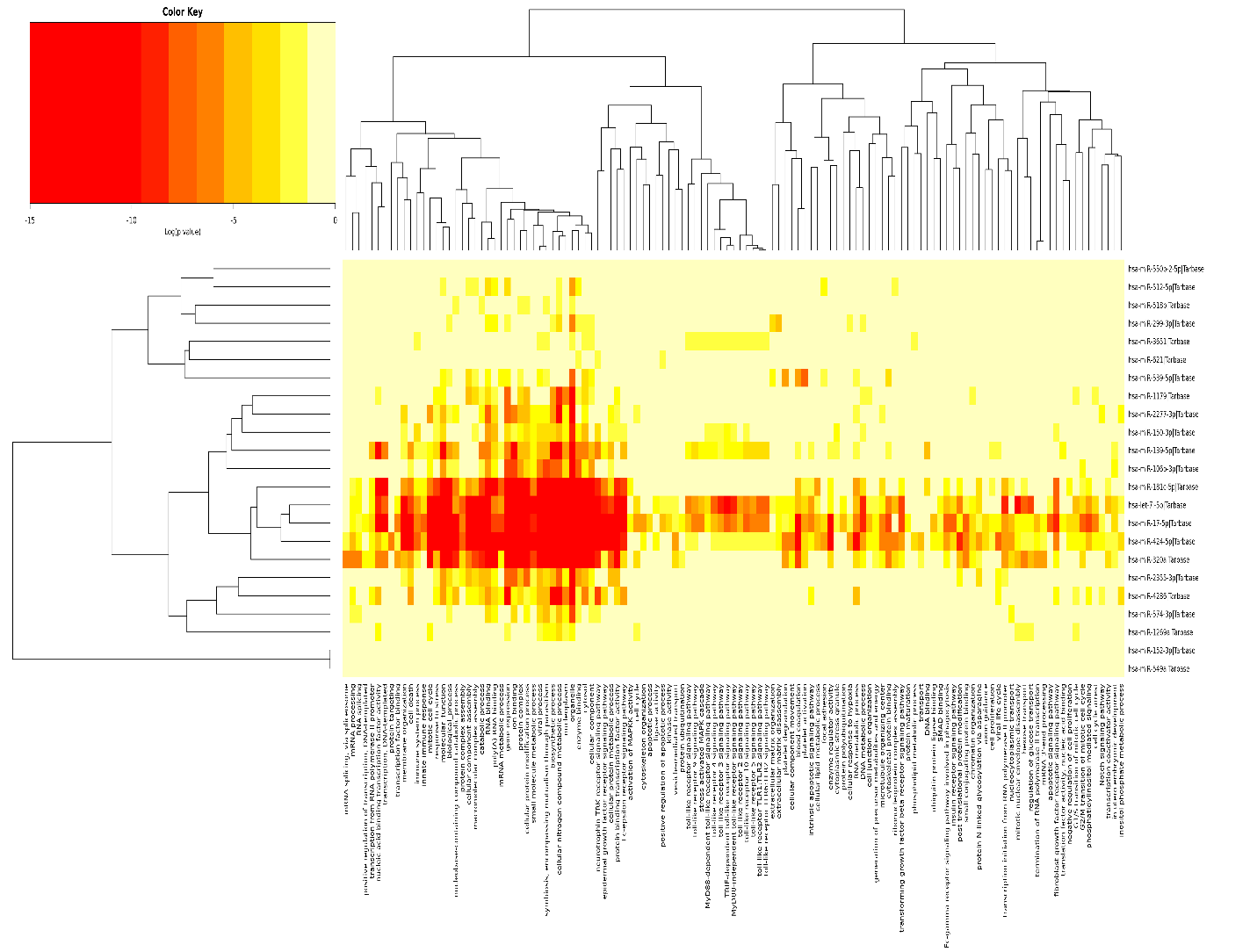


**Supplementary Figure S5**. Heatmap showing enrichment of 23-miRNA signature in GO annotations


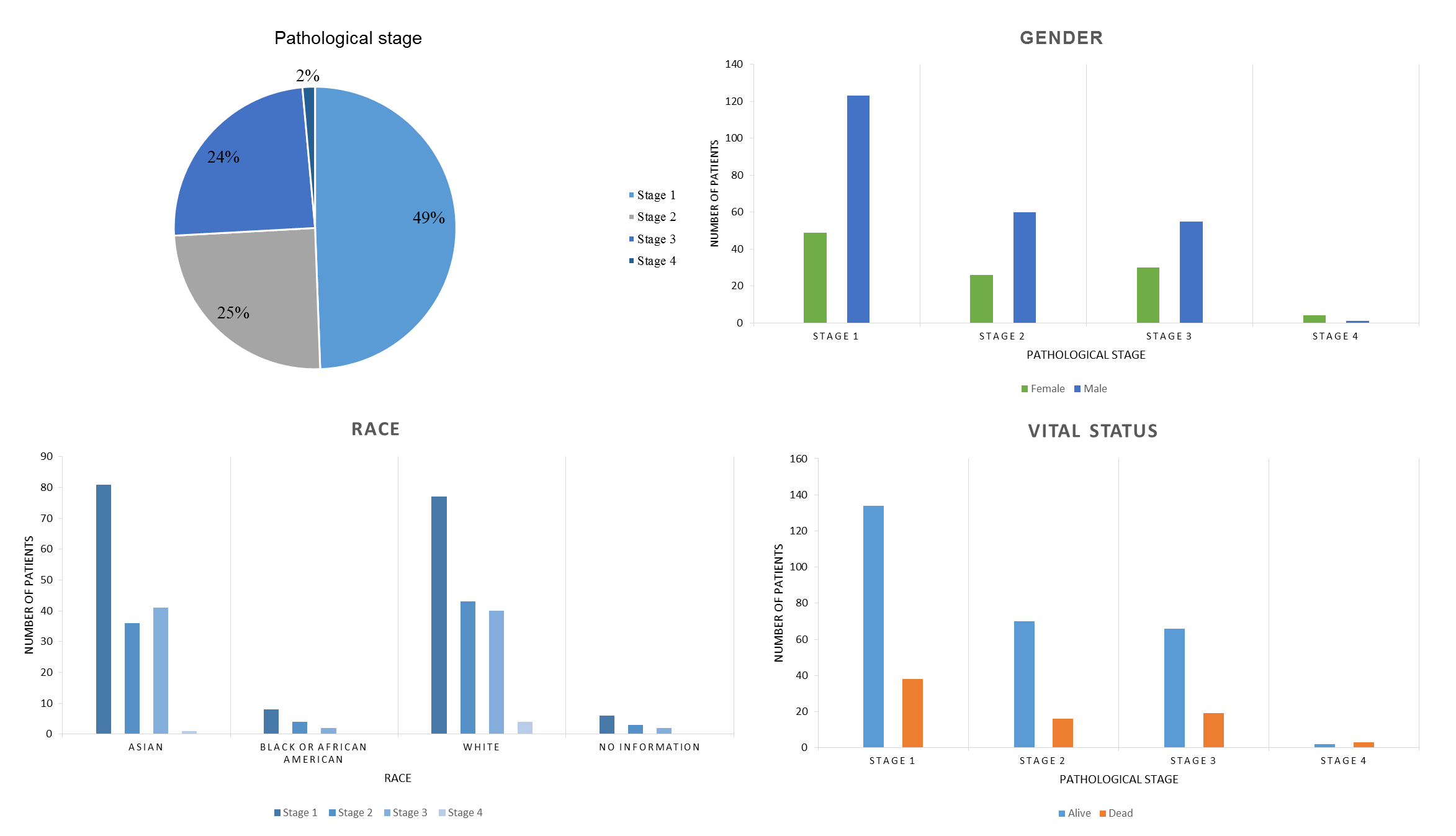


**Supplementary Figure S6**. The clinical characterization of patients with hepatocellular carcinoma (n=348)

.

# Supplementary Tables

**Supplementary Table S1**. Prediction performance of the machine learning methods using different feature numbers.

| **Method** | **Training accuracy (%)** | **Selected miRNAs** | **Sensitivity** | **Specificity** | **MCC** | **AUC** |
| --- | --- | --- | --- | --- | --- | --- |
| SMO | 73.85 | 19 | 0.99 | 0.004 | 0.03 | 0.5 |
| MLP | 67.52 | 19 | 0.79 | 0.2 | 0.13 | 0.6 |
| Naïve Bayes | 74.13 | 19 | 0.81 | 0.46 | 0.34 | 0.71 |
| LibSVM | 77.58 | 19 | 0.97 | 0.02 | 0.3 | 0.58 |
| Random forest | 76.43 | 19 | 0.94 | 0.58 | 0.27 | 0.73 |

**Supplementary Table S2**. The statistical analysis of comparing AUCs of machine learning methods with SVM-HCC.

| **Method** | **AUC** | **Standard error** | **Difference (AUC*_SVM-HCC_* -COM*)** | **Z score** | **P-value** |
| --- | --- | --- | --- | --- | --- |
| SMO | 0.50 | 0.030 | 0.4 | 10.29 | <0.001 |
| MLP | 0.60 | 0.033 | 0.3 | 8.099 | <0.001 |
| Naïve Bayes | 0.71 | 0.029 | 0.19 | 5.71 | <0.001 |
| LibSVM | 0.54 | 0.034 | 0.36 | 9.34 | <0.001 |
| Random forest | 0.72 | 0.028 | 0.18 | 5.48 | <0.001 |
| SVM-HCC | 0.90 | 0.016 | 0 | 0 | 1 |

* Comparison of other methods (COM)

**Supplementary Table S3**. The expression difference of the 10 top-ranked miRNAs in the normal and tumor groups (*t*-test).

| **miRNA** | **Normal vs Tumor (p-value)** |
| --- | --- |
| hsa-miR-550a | 6.74E-03 |
| hsa-miR-549 | NA |
| hsa-miR-518b | 2.17E-03 |
| hsa-miR-512 | 1.65E-03 |
| hsa-miR-1179 | 1.71E-01 |
| hsa-miR-574 | 1.47E-12 |
| hsa-miR-424 | 1.62E-12 |
| hsa-miR-4286 | 5.35E-03 |
| hsa-let-7i | 1.16E-02 |
| hsa-miR-320a | 4.61E-09 |

**Supplementary Table S4**. Summary of 10 top-ranked miRNAs involved in HCC and other cancers.

| **Rank** | **miRNA** | **Regulation** | **Cancer** | **Reference** |
| --- | --- | --- | --- | --- |
| 1 | hsa-miR-550a | Up | HCC | (1), |
|  |  |  | non-small cell lung cancer, | (2) |
|  |  | Down | Brest cancer | (3) |
| 2 | Hsa-miR-549 | Up | colorectal cancer, breast cancer, | (4-6) |
| 3 | hsa-miR-518b | Up | HCC | (7), (8) |
| 4 | hsa-miR-512 | Up | HCC | (7), (9) |
| 5 | hsa-miR-1179 | Down  Up | HCC  esophageal squamous cell carcinoma | (10), (11)  (12) |
| 6 | hsa-miR-574 | Up | HCC | (13) |
| 7 | hsa-miR-424 | Down | HCC | (14) |
| 8 | hsa-miR-4286 | Down | HCC | (15-17) |
| 9 | Hsa-let-7i | Up | HCC | (18) |
| 10 | hsa-miR-320a | Up | HCC | (19), (20) |

**Supplementary Table S5**. Gene Ontology annotations of 10 top-ranked miRNAs.

|  | **GO Category** | **Genes** | **MiRNAs** | **Adjusted p-value** |
| --- | --- | --- | --- | --- |
| **Biological pathways** | mitotic cell cycle | 156 | 3 | <0.001 |
|  | blood coagulation | 122 | 3 | <0.001 |
|  | cellular protein metabolic process | 146 | 3 | <0.001 |
|  | membrane organization | 182 | 3 | <0.001 |
|  | epidermal growth factor receptor signaling pathway | 68 | 4 | <0.001 |
|  | cell death | 259 | 4 | <0.001 |
|  | Fc-epsilon receptor signaling pathway | 55 | 4 | <0.001 |
|  | small molecule metabolic process | 604 | 4 | <0.001 |
|  | response to stress | 557 | 5 | <0.001 |
|  | nucleobase-containing compound catabolic process | 265 | 5 | <0.001 |
| **Molecular functions** | protein binding transcription factor activity | 185 | 5 | <0.001 |
|  | nucleic acid binding transcription factor activity | 251 | 5 | <0.001 |
|  | ion binding | 1446 | 5 | <0.001 |
|  | RNA binding | 592 | 7 | <0.001 |
|  | enzyme binding | 420 | 7 | <0.001 |
|  | poly(A) RNA binding | 493 | 7 | <0.001 |
|  | cytoskeletal protein binding | 199 | 3 | <0.001 |
|  | enzyme regulator activity | 209 | 3 | <0.001 |
|  | small conjugating protein binding | 30 | 2 | <0.001 |
|  | transcription factor binding | 105 | 2 | <0.001 |
| **Cellular components** | cytosol | 830 | 6 | <0.001 |
|  | protein complex | 1059 | 6 | <0.001 |
|  | nucleoplasm | 437 | 7 | <0.001 |
|  | organelle | 2729 | 8 | <0.001 |
|  | microtubule organizing center | 126 | 3 | <0.001 |
|  | focal adhesion | 92 | 2 | 0.001 |

**Supplementary Table S6**. 23-miRNA signature and co-expressed miRNAs with R≥0.5.

| **MiRNA** | **Co-expressed miRNA** | **Correlation (R)** |
| --- | --- | --- |
| hsa-miR-518b | hsa-miR-512 | 0.883679393 |
|  | hsa-miR-525 | 0.881350065 |
|  | hsa-miR-517 | 0.88 |
|  | hsa-miR-520a | 0.870766301 |
|  | hsa-miR-526b | 0.86433142 |
|  | hsa-miR-519a | 0.859178843 |
|  | hsa-miR-516a | 0.844806103 |
|  | hsa-miR-522 | 0.842625455 |
|  | hsa-miR-1323 | 0.807107576 |
| hsa-miR-512 | hsa-miR-518b | 0.871746257 |
|  | hsa-miR-525 | 0.865907046 |
|  | hsa-miR-520b | 0.859927914 |
|  | hsa-miR-519a | 0.856579876 |
|  | hsa-miR-522 | 0.852744827 |
|  | hsa-miR-516a | 0.843403559 |
|  | hsa-miR-526b | 0.841374515 |
|  | hsa-miR-517a | 0.836467926 |
|  | hsa-miR-1323 | 0.829066303 |
| hsa-miR-424 | hsa-miR-542 | 0.777401805 |
|  | hsa-miR-450a | 0.687428987 |
|  | hsa-miR-214 | 0.655559853 |
|  | hsa-miR-199a | 0.635275976 |
|  | hsa-miR-143 | 0.612333694 |
|  | hsa-miR-10a | 0.605803688 |
|  | hsa-miR-145 | 0.603392878 |
|  | hsa-miR-218.2 | 0.598560623 |
|  | hsa-miR-27a | 0.575610793 |
|  | hsa-miR-99b | 0.552058696 |
|  | hsa-miR-23a | 0.550158598 |
|  | hsa-miR-125a | 0.541655412 |
|  | hsa-let-7i | 0.529601998 |
|  | hsa-miR-142 | 0.529210797 |
|  | hsa-miR-181c | 0.507742953 |
| hsa-let-7i | hsa-miR-98 | 0.599950303 |
|  | hsa-miR-342 | 0.585153104 |
|  | hsa-miR-708 | 0.578012687 |
|  | hsa-let-7b | 0.573589724 |
|  | hsa-miR-199a | 0.572399645 |
|  | hsa-miR-214 | 0.559081365 |
|  | hsa-miR-218.2 | 0.553461517 |
|  | hsa-miR-10a | 0.546107514 |
|  | hsa-miR-142 | 0.545629582 |
|  | hsa-miR-598 | 0.540115603 |
|  | hsa-miR-424 | 0.529601998 |
|  | hsa-miR-155 | 0.527011231 |
|  | hsa-miR-125a | 0.523630675 |
|  | hsa-miR-145 | 0.52022292 |
|  | hsa-miR-542 | 0.506432197 |
|  | hsa-miR-450a | 0.501729332 |
| hsa-miR-320a | hsa-miR-1301 | 0.585927941 |

**Supplementary Table S7**. Involvement of hsa-miR-518, hsa-miR-512, and their co-expressed miRNAs in KEGG pathways.

| **MiRNA** | **KEGG pathway** | **MiRNAs** | **Genes** | **Ensembl-ID** | **Adjusted p-value** |
| --- | --- | --- | --- | --- | --- |
| hsa-miR-518 | Glycosphingolipid biosynthesis - lacto and neolacto series (hsa00601) | hsa-miR-525-5p | FUT1 | ENSG00000174951 | <0.001 |
|  |  |  |  |  |  |
|  |  | hsa-miR-517-5p | FUT1 | ENSG00000174951 | <0.001 |
|  |  | hsa-miR-516a-3p | FUT4 | ENSG00000196371 | <0.001 |
|  | Folate biosynthesis (hsa00790) | hsa-miR-525-5p | MOCS1 | ENSG00000124615 | <0.05 |
|  |  | hsa-miR-525-5p | DHFR | ENSG00000228716 | <0.05 |
|  |  | hsa-miR-522-3p | DHFRL1 | ENSG00000178700 | <0.001 |
|  | One carbon pool by folate (hsa00670) | hsa-miR-522-3p | DHFR | ENSG00000228716 | <0.001 |
|  |  |  | DHFRL1 | ENSG00000178700 | <0.001 |
|  | Mucin type O-Glycan biosynthesis (hsa00512) | hsa-miR-517-5p | GCNT1 | ENSG00000187210 | <0.05 |
|  |  | hsa-miR-520a-3p | GCNT1 | ENSG00000187210 | <0.001 |
| hsa-miR-512 | Central carbon metabolism in cancer (hsa05230) | hsa-miR-526b-5p | PKM | ENSG00000067225 | <0.001 |
|  |  | hsa-miR-516a-5p | SLC16A3 | ENSG00000141526 | <0.001 |
|  |  |  | MYC | ENSG00000136997 | <0.001 |
|  |  | hsa-miR-519a-5p | AKT3 | ENSG00000117020 | <0.001 |
|  |  |  | PTEN | ENSG00000171862 | <0.001 |
|  |  | hsa-miR-520b | PFKP | ENSG00000067057 | <0.001 |

**Supplementary Table S8.** Involvement of hsa-miR-424 and its co-expressed miRNAs in cancer and other pathways.

|  | **KEGG pathway** | **miRNA** | | **No.of target genes** | **Adjusted p-value** |
| --- | --- | --- | --- | --- | --- |
| 1 | Prion diseases (hsa05020) | hsa-miR-542-3p, hsa-miR-23a-3p | 7 | | <0.001 |
| 2 | Fatty acid biosynthesis (hsa00061) | hsa-miR-10a-5p, hsa-miR-218-5p, hsa-miR-23a-3p, hsa-miR-125a-5p | 2 | | <0.001 |
| 3 | ECM-receptor interaction (hsa04512) | hsa-miR-10a-5p, hsa-miR-218-5p, hsa-miR-23a-3p, hsa-miR-125a-5p | 20 | | <0.001 |
| 4 | Proteoglycans in cancer (hsa05205) | hsa-miR-542-3p, hsa-miR-199a-5p, hsa-miR-218-5p, hsa-miR-27a-5p, hsa-miR-23a-3p, hsa-miR-125a-5p | 88 | | <0.001 |
| 5 | Hippo signaling pathway (hsa04390) | hsa-miR-199a-5p, hsa-miR-10a-5p, hsa-miR-218-5p, hsa-miR-27a-5p, hsa-miR-99b-3p, hsa-miR-125a-5p | 59 | | <0.001 |
| 6 | Fatty acid metabolism (hsa01212) | hsa-miR-10a-5p, hsa-miR-218-5p, hsa-miR-27a-5p, hsa-miR-23a-3p, hsa-miR-125a-5p | 14 | | <0.001 |
| 7 | Adherens junction (hsa04520) | hsa-miR-542-3p, hsa-miR-199a-5p, hsa-miR-218-5p, hsa-miR-27a-5p, hsa-miR-99b-3p, hsa-miR-23a-3p, hsa-miR-125a-5p | 43 | | <0.001 |
| 8 | Viral carcinogenesis (hsa05203) | hsa-miR-424-3p, hsa-miR-542-3p, hsa-miR-218-5p, hsa-miR-23a-3p, hsa-miR-125a-5p, hsa-miR-142-5p | 80 | | <0.001 |
| 9 | Biotin metabolism (hsa00780) | hsa-miR-218-5p, hsa-let-7i-3p, | 1 | | <0.001 |
| 10 | Pathways in cancer (hsa05200) | hsa-miR-218-5p, hsa-miR-27a-5p, hsa-miR-23a-3p | 115 | | <0.001 |
| 11 | Cell cycle (hsa04110) | hsa-miR-424-3p, hsa-miR-542-3p, hsa-miR-99b-3p, hsa-miR-23a-3p, hsa-miR-125a-5p, hsa-miR-142-5p | 46 | | <0.001 |
| 12 | p53 signaling pathway (hsa04115) | hsa-miR-218-5p, hsa-miR-23a-3p, hsa-miR-142-5p | 34 | | <0.05 |
| 13 | Other types of O-glycan biosynthesis (hsa00514) | hsa-miR-450a-5p, hsa-miR-27a-5p, hsa-miR-125a-5p, hsa-miR-181c-3p | 8 | | <0.05 |
| 14 | Steroid biosynthesis (hsa00100) | hsa-miR-23a-3p, hsa-let-7i-3p | 3 | | <0.05 |
| 15 | Glioma (hsa05214) | hsa-miR-218-5p, hsa-miR-27a-5p, hsa-miR-23a-3p, hsa-miR-142-5p | 30 | | <0.05 |

**Supplementary Table S9**. Involvement of hsa-let-7i and its co-expressed miRNAs in cancer and other pathways.

| **KEGG pathway** | **MiRNAs** | **Genes** | **Adjusted p-value** |
| --- | --- | --- | --- |
| Proteoglycans in cancer | 14 | 102 | <0.001 |
| Hepatitis B | 14 | 77 | <0.001 |
| Cell cycle | 14 | 69 | <0.001 |
| TGF-beta signaling pathway | 15 | 44 | <0.001 |
| Renal cell carcinoma | 11 | 41 | <0.001 |
| Adherens junction | 14 | 46 | <0.001 |
| Lysine degradation | 11 | 25 | <0.001 |
| Viral carcinogenesis | 15 | 91 | <0.001 |
| Hippo signaling pathway | 14 | 73 | <0.001 |
| TNF signaling pathway | 12 | 62 | <0.001 |
| Fatty acid biosynthesis | 5 | 4 | <0.001 |
| Chronic myeloid leukemia | 13 | 42 | <0.001 |
| Transcriptional misregulation in cancer | 15 | 75 | <0.001 |
| ECM-receptor interaction | 13 | 32 | <0.001 |
| FoxO signaling pathway | 14 | 68 | 0.001 |
| HIF-1 signaling pathway | 13 | 55 | 0.001 |
| Pathways in cancer | 15 | 160 | 0.001 |
| RNA transport | 13 | 80 | 0.001 |
| p53 signaling pathway | 12 | 40 | <0.05 |
| Bladder cancer | 12 | 25 | <0.05 |
| Colorectal cancer | 13 | 35 | <0.05 |
| Glioma | 12 | 33 | <0.05 |
| Prostate cancer | 14 | 48 | <0.05 |
| Pancreatic cancer | 12 | 37 | <0.05 |
| Protein processing in endoplasmic reticulum | 14 | 78 | <0.05 |
| Estrogen signaling pathway | 13 | 45 | <0.05 |
| Focal adhesion | 15 | 95 | <0.05 |
| Thyroid cancer | 11 | 17 | <0.05 |
| Ubiquitin mediated proteolysis | 15 | 69 | <0.05 |
| Central carbon metabolism in cancer | 13 | 31 | <0.05 |
| Endocytosis | 14 | 90 | <0.05 |
| HTLV-I infection | 14 | 108 | <0.05 |
| Thyroid hormone signaling pathway | 15 | 55 | <0.05 |
| Oocyte meiosis | 14 | 51 | <0.05 |
| Steroid biosynthesis | 9 | 10 | <0.05 |
| Endometrial cancer | 13 | 27 | <0.05 |
| Arrhythmogenic right ventricular cardiomyopathy (ARVC) | 13 | 26 | <0.05 |
| Chagas disease (American trypanosomiasis) | 13 | 49 | 0.05 |
| Melanoma | 12 | 33 | 0.05 |
| Small cell lung cancer | 13 | 42 | 0.06 |
| Fatty acid metabolism | 10 | 16 | 0.05 |
| Bacterial invasion of epithelial cells | 14 | 37 | 0.08 |
| MAPK signaling pathway | 15 | 103 | 0.09 |
| Neurotrophin signaling pathway | 13 | 54 | 0.12 |
| Insulin signaling pathway | 15 | 63 | 0.14 |
| Epstein-Barr virus infection | 15 | 87 | 0.15 |
| AMPK signaling pathway | 15 | 59 | 0.16 |
| PI3K-Akt signaling pathway | 15 | 136 | 0.25 |
| Base excision repair | 10 | 17 | 0.19 |
| Non-small cell lung cancer | 13 | 26 | 0.25 |
| RNA degradation | 14 | 37 | 0.27 |
| Shigellosis | 14 | 33 | 0.30 |
| Biosynthesis of unsaturated fatty acids | 6 | 9 | 0.14 |
| Apoptosis | 12 | 39 | 0.30 |
| Sphingolipid signaling pathway | 13 | 48 | 0.34 |
| Epithelial cell signaling in Helicobacter pylori infection | 13 | 32 | 0.35 |
| Signaling pathways regulating pluripotency of stem cells | 15 | 55 | 0.45 |
| mTOR signaling pathway | 14 | 30 | 0.43 |
| Prolactin signaling pathway | 12 | 34 | 0.41 |
| Acute myeloid leukemia | 11 | 26 | 0.43 |

**Supplementary Table S10.** Tarbase experimentally supported gene interactions for hsa-let-7i and its co-expressed miRNAs involved in the hepatitis B pathway (Bonferroni adjusted p-value<0.001).

| **MiRNA** | **Gene Name** | **Gene Ensembl id** |
| --- | --- | --- |
| hsa-miR-145-5p | PRKCA | ENSG00000154229 |
|  | STAT3 | ENSG00000168610 |
|  | CDK4 | ENSG00000135446 |
|  | DDX3X | ENSG00000215301 |
|  | TGFB1 | ENSG00000105329 |
|  | CCND1 | ENSG00000110092 |
|  | AKT1 | ENSG00000142208 |
|  | MYC | ENSG00000136997 |
|  | MMP9 | ENSG00000100985 |
|  | TGFB2 | ENSG00000092969 |
|  | TGFB3 | ENSG00000119699 |
| hsa-miR-10a-3p | FOS | ENSG00000170345 |
|  | TGFBR1 | ENSG00000106799 |
|  | CXCL8 | ENSG00000169429 |
|  | CHUK | ENSG00000213341 |
|  | CDK6 | ENSG00000105810 |
|  | DDX3X | ENSG00000215301 |
|  | CREB1 | ENSG00000118260 |
|  | MAPK9 | ENSG00000050748 |
|  | CCND1 | ENSG00000110092 |
|  | SMAD4 | ENSG00000141646 |
|  | IRF3 | ENSG00000126456 |
|  | EP300 | ENSG00000100393 |
| hsa-let-7b-5p | TGFBR1 | ENSG00000106799 |
|  | ATF2 | ENSG00000115966 |
|  | NRAS | ENSG00000213281 |
|  | MAP2K2 | ENSG00000126934 |
|  | CCNA2 | ENSG00000145386 |
|  | ATF6B | ENSG00000213676 |
|  | CDK2 | ENSG00000123374 |
|  | BAX | ENSG00000087088 |
|  | CHUK | ENSG00000213341 |
|  | MAP3K1 | ENSG00000095015 |
|  | TLR4 | ENSG00000136869 |
|  | CDK6 | ENSG00000105810 |
|  | DDX3X | ENSG00000215301 |
|  | CCNA1 | ENSG00000133101 |
|  | TP53 | ENSG00000141510 |
|  | HSPG2 | ENSG00000142798 |
|  | APAF1 | ENSG00000120868 |
|  | MAVS | ENSG00000088888 |
|  | CASP3 | ENSG00000164305 |
|  | CCND1 | ENSG00000110092 |
|  | CCNE2 | ENSG00000175305 |
|  | E2F3 | ENSG00000112242 |
|  | MAPK8 | ENSG00000107643 |
|  | AKT1 | ENSG00000142208 |
|  | MYC | ENSG00000136997 |
|  | IRF3 | ENSG00000126456 |
|  | RB1 | ENSG00000139687 |
|  | YWHAZ | ENSG00000164924 |
|  | TBK1 | ENSG00000183735 |
|  | FAS | ENSG00000026103 |
|  | CREB3L2 | ENSG00000182158 |
|  | PIK3CA | ENSG00000121879 |
|  | CDKN1A | ENSG00000124762 |
|  | MAP2K4 | ENSG00000065559 |
|  | NFATC3 | ENSG00000072736 |
|  | MAPK1 | ENSG00000100030 |
|  | JAK1 | ENSG00000162434 |
|  | ATF4 | ENSG00000128272 |
| hsa-let-7i-5p | STAT3 | ENSG00000168610 |
|  | TGFBR1 | ENSG00000106799 |
|  | CDK4 | ENSG00000135446 |
|  | ATF2 | ENSG00000115966 |
|  | NRAS | ENSG00000213281 |
|  | CCNA2 | ENSG00000145386 |
|  | ATF6B | ENSG00000213676 |
|  | CREB5 | ENSG00000146592 |
|  | CDK2 | ENSG00000123374 |
|  | CHUK | ENSG00000213341 |
|  | MAP3K1 | ENSG00000095015 |
|  | TLR4 | ENSG00000136869 |
|  | CDK6 | ENSG00000105810 |
|  | DDX3X | ENSG00000215301 |
|  | TP53 | ENSG00000141510 |
|  | HSPG2 | ENSG00000142798 |
|  | APAF1 | ENSG00000120868 |
|  | MAVS | ENSG00000088888 |
|  | CASP3 | ENSG00000164305 |
|  | CCND1 | ENSG00000110092 |
|  | CCNE2 | ENSG00000175305 |
|  | E2F3 | ENSG00000112242 |
|  | MAPK8 | ENSG00000107643 |
|  | MYC | ENSG00000136997 |
|  | IRF3 | ENSG00000126456 |
|  | RB1 | ENSG00000139687 |
|  | YWHAZ | ENSG00000164924 |
|  | TBK1 | ENSG00000183735 |
|  | FAS | ENSG00000026103 |
|  | CREB3L2 | ENSG00000182158 |
|  | CREB3L1 | ENSG00000157613 |
|  | CDKN1A | ENSG00000124762 |
|  | MAP2K4 | ENSG00000065559 |
|  | NFATC3 | ENSG00000072736 |
|  | MAPK1 | ENSG00000100030 |
|  | JAK1 | ENSG00000162434 |
|  | ATF4 | ENSG00000128272 |
| hsa-miR-155-5p | STAT3 | ENSG00000168610 |
|  | NFKB1 | ENSG00000109320 |
|  | CDK4 | ENSG00000135446 |
|  | E2F2 | ENSG00000007968 |
|  | CDK2 | ENSG00000123374 |
|  | SMAD3 | ENSG00000166949 |
|  | BCL2 | ENSG00000171791 |
|  | DDB2 | ENSG00000134574 |
|  | KRAS | ENSG00000133703 |
|  | MAVS | ENSG00000088888 |
|  | MYD88 | ENSG00000172936 |
|  | CCND1 | ENSG00000110092 |
|  | SMAD4 | ENSG00000141646 |
|  | E2F3 | ENSG00000112242 |
|  | PIK3R1 | ENSG00000145675 |
|  | YWHAZ | ENSG00000164924 |
|  | AKT3 | ENSG00000117020 |
|  | CDKN1A | ENSG00000124762 |
|  | STAT1 | ENSG00000115415 |
|  | TNF | ENSG00000232810 |
|  | RELA | ENSG00000173039 |
|  | IL6 | ENSG00000136244 |
| hsa-miR-142-5p | CDKN1B | ENSG00000111276 |
|  | MAP3K1 | ENSG00000095015 |
|  | KRAS | ENSG00000133703 |
|  | CDK6 | ENSG00000105810 |
|  | MAVS | ENSG00000088888 |
|  | CCND1 | ENSG00000110092 |
|  | DDB1 | ENSG00000167986 |
|  | EP300 | ENSG00000100393 |
|  | CASP8 | ENSG00000064012 |
|  | CCNE1 | ENSG00000105173 |
|  | PTEN | ENSG00000171862 |
|  | MAPK1 | ENSG00000100030 |
| hsa-miR-125a-3p | CDKN1B | ENSG00000111276 |
|  | CDK6 | ENSG00000105810 |
|  | SMAD4 | ENSG00000141646 |
|  | MYC | ENSG00000136997 |
|  | MAPK3 | ENSG00000102882 |
|  | EP300 | ENSG00000100393 |
|  | PIK3CA | ENSG00000121879 |
|  | IL6 | ENSG00000136244 |
|  | MAPK1 | ENSG00000100030 |
|  | STAT4 | ENSG00000138378 |
|  | ELK1 | ENSG00000126767 |
| hsa-miR-199a-5p | PRKCA | ENSG00000154229 |
|  | TGFBR1 | ENSG00000106799 |
|  | YWHAB | ENSG00000166913 |
|  | KRAS | ENSG00000133703 |
|  | DDX3X | ENSG00000215301 |
|  | TGFB1 | ENSG00000105329 |
|  | JUN | ENSG00000177606 |
|  | SMAD4 | ENSG00000141646 |
|  | MYC | ENSG00000136997 |
| hsa-miR-214-3p | STAT3 | ENSG00000168610 |
|  | NRAS | ENSG00000213281 |
|  | TP53 | ENSG00000141510 |
|  | HSPG2 | ENSG00000142798 |
|  | PTEN | ENSG00000171862 |
|  | MAPK1 | ENSG00000100030 |
|  | JAK1 | ENSG00000162434 |
|  | ATF4 | ENSG00000128272 |
| hsa-miR-424-3p | CCNA2 | ENSG00000145386 |
|  | CDK2 | ENSG00000123374 |
|  | CDK6 | ENSG00000105810 |
|  | PTEN | ENSG00000171862 |
| hsa-miR-708-3p | NRAS | ENSG00000213281 |
|  | CASP3 | ENSG00000164305 |
|  | MAPK3 | ENSG00000102882 |
|  | MAPK1 | ENSG00000100030 |
| hsa-miR-542-5p | HSPG2 | ENSG00000142798 |
| hsa-miR-342-5p | AKT2 | ENSG00000105221 |
|  | DDB1 | ENSG00000167986 |
| hsa-miR-450a-5p | CXCL8 | ENSG00000169429 |
|  | CDKN1B | ENSG00000111276 |

**Supplementary Table S11**. Involvement of hsa-miR-320 and its co-expressed miRNAs in KEGG pathways.

| **KEGG pathway** | **Genes** | **Adjusted p-value** |
| --- | --- | --- |
| TGF-beta signaling pathway | 13 | <0.001 |
| Hippo signaling pathway | 20 | <0.001 |
| Adherens junction | 12 | <0.001 |
| Estrogen signaling pathway | 15 | <0.05 |
| Transcriptional misregulation in cancer | 26 | <0.05 |
| Sulfur relay system | 2 | <0.05 |
| Glioma | 11 | 0.05 |
| Long-term depression | 9 | 0.07135 |
| Cocaine addiction | 6 | 0.07186 |
| Colorectal cancer | 11 | 0.13175 |
| Chronic myeloid leukemia | 14 | 0.16768 |
| Viral carcinogenesis | 27 | 0.18877 |
| Pancreatic cancer | 11 | 0.38203 |
| Signaling pathways regulating pluripotency of stem cells | 18 | 0.41748 |
| Pathways in cancer | 42 | 0.42722 |
| Spliceosome | 18 | 0.5424 |

**Supplementary Table S12**. Correlation between 23-miRNA signature and clinicopathological features

| **miRNA** | **Measurement** | **Ethnicity** | **Gender** | **Height** | **Race** | **Viral hepatitis infection** | **Vital status** | **Weight** |
| --- | --- | --- | --- | --- | --- | --- | --- | --- |
| hsa-miR-550a | Correlation Coefficient | .152** | -0.096 | -0.005 | 0.055 | 0.067 | 0.048 | -0.009 |
|  | Sig. (2-tailed) | 0.003 | 0.063 | 0.912 | 0.284 | 0.196 | 0.351 | 0.855 |
| hsa-miR-549 | Correlation Coefficient | 0.005 | -0.075 | -0.044 | -0.039 | 0.032 | 0.0154 | -0.034 |
|  | Sig. (2-tailed) | 0.911 | 0.144 | 0.394 | 0.448 | 0.530 | 0.766 | 0.504 |
| hsa-miR-518b | Correlation Coefficient | -0.011 | -0.017 | 0.039 | 0.069 | 0.070 | -0.025 | 0.089 |
|  | Sig. (2-tailed) | 0.829 | 0.738 | 0.446 | 0.180 | 0.177 | 0.627 | 0.086 |
| hsa-miR-512-2 | Correlation Coefficient | -0.072 | 0.052 | 0.081 | 0.060 | 0.065 | -0.052 | 0.066 |
|  | Sig. (2-tailed) | 0.163207 | 0.31105 | 0.117545 | 0.24263 | 0.210676 | 0.314419 | 0.202962 |
| hsa-miR-1179 | Correlation Coefficient | -0.004 | -0.080 | -0.051 | 0.071 | 0.028 | .122* | 0.015 |
|  | Sig. (2-tailed) | 0.938 | 0.122 | 0.318 | 0.168 | 0.578 | 0.018 | 0.763 |
| hsa-miR-574 | Correlation Coefficient | 0.028 | -0.049 | -0.001 | 0.031 | 0.095 | 0.005 | 0.044 |
|  | Sig. (2-tailed) | 0.585 | 0.340 | 0.980 | 0.548 | 0.066 | 0.920 | 0.389 |
| hsa-miR-424 | Correlation Coefficient | -0.045 | -0.019 | 0.050 | 0.067 | 0.084 | 0.053 | 0.076 |
|  | Sig. (2-tailed) | 0.380 | 0.705 | 0.328 | 0.194 | 0.105 | 0.304 | 0.142 |
| hsa-miR-4286 | Correlation Coefficient | -0.1 | -0.017 | 0.013 | 0.047 | 0.047 | 0.014 | -0.024 |
|  | Sig. (2-tailed) | 0.053 | 0.743 | 0.799 | 0.358 | 0.363 | 0.773 | 0.639 |
| hsa-let-7i | Correlation Coefficient | -0.040 | 0.028 | 0.057 | 0.100 | .167** | 0.0008 | .102* |
|  | Sig. (2-tailed) | 0.434 | 0.588 | 0.265 | 0.053 | 0.001 | 0.987 | 0.048 |
| hsa-miR-320a | Correlation Coefficient | -0.017 | -0.068 | -0.029 | 0.020 | .190** | -0.086 | -0.007 |
|  | Sig. (2-tailed) | 0.739 | 0.184 | 0.570 | 0.696 | 0.0002 | 0.094 | 0.889 |
| hsa-miR-17 | Correlation Coefficient | -0.085 | 0.032 | -0.017 | -.138** | -0.065 | -.196** | -0.053 |
|  | Sig. (2-tailed) | 0.100 | 0.532 | 0.741 | 0.007 | 0.205 | 0.0001 | 0.305 |
| hsa-miR-299 | Correlation Coefficient | -0.059 | -0.065 | 0.024 | 0.042 | -0.008 | 0.021 | 0.053 |
|  | Sig. (2-tailed) | 0.254 | 0.209 | 0.637 | 0.415 | 0.869 | 0.674 | 0.303 |
| hsa-miR-3651 | Correlation Coefficient | -0.096 | 0.044 | 0.068 | 0.027 | 0.086 | 0.017 | 0.069 |
|  | Sig. (2-tailed) | 0.0625 | 0.389 | 0.187 | 0.591 | 0.096 | 0.731 | 0.180 |
| hsa-miR-2277 | Correlation Coefficient | -0.034 | 0.0389 | 0.0758 | 0.061 | 0.021 | 0.044 | 0.012 |
|  | Sig. (2-tailed) | 0.509 | 0.453 | 0.144 | 0.237 | 0.677 | 0.387 | 0.804 |
| hsa-miR-621 | Correlation Coefficient | 0.030 | -0.009 | -0.004 | -0.025 | 0.020 | .129* | -0.076 |
|  | Sig. (2-tailed) | 0.562 | 0.856 | 0.928 | 0.618 | 0.696 | 0.012 | 0.140 |
| hsa-miR-181c | Correlation Coefficient | -0.082 | -0.016 | 0.058 | -0.068 | 0.012 | -0.089 | -0.058 |
|  | Sig. (2-tailed) | 0.113 | 0.744 | 0.258 | 0.185 | 0.813 | 0.083 | 0.263 |
| hsa-miR-539 | Correlation Coefficient | -.103* | -0.025 | 0.022 | 0.013 | 0.018 | 0.048 | -0.001 |
|  | Sig. (2-tailed) | 0.046 | 0.622 | 0.66 | 0.792 | 0.728 | 0.350 | 0.981 |
| hsa-miR-106b | Correlation Coefficient | 0.024 | -0.0002 | 0.018 | -0.067 | 0.059 | -0.085 | 0.001 |
|  | Sig. (2-tailed) | 0.637 | 0.996 | 0.722 | 0.190 | 0.249 | 0.101 | 0.973 |
| hsa-miR-1269 | Correlation Coefficient | .107* | -0.079 | -0.012 | 0.074 | -0.022 | 0.056 | 0.040 |
|  | Sig. (2-tailed) | 0.038 | 0.124 | 0.809 | 0.152 | 0.671 | 0.278 | 0.431 |
| hsa-miR-139 | Correlation Coefficient | -0.092 | -0.005 | -0.002 | -0.033 | -0.035 | 0.039 | -0.045 |
|  | Sig. (2-tailed) | 0.075 | 0.923 | 0.960 | 0.521 | 0.500 | 0.442 | 0.380 |
| hsa-miR-152 | Correlation Coefficient | 0.059 | -0.021 | -0.053 | -0.040 | -0.074 | 0.074 | 0.032 |
|  | Sig. (2-tailed) | 0.252 | 0.686 | 0.302 | 0.438 | 0.153 | 0.149 | 0.528 |
| hsa-miR-2355 | Correlation Coefficient | 0.070 | 0.0003 | .174** | 0.071 | .156** | -0.101 | .109* |
|  | Sig. (2-tailed) | 0.172 | 0.994 | 0.0007 | 0.170 | 0.002 | 0.050 | 0.035 |
| hsa-miR-150 | Correlation Coefficient | -0.024 | 0.078 | 0.026 | -0.014 | 0.014 | -.164** | .124* |
|  | Sig. (2-tailed) | 0.639 | 0.130 | 0.612 | 0.781 | 0.775 | 0.001 | 0.017 |

**. Correlation is significant at the 0.01 level (2-tailed).

*. Correlation is significant at the 0.05 level (2-tailed).

**Supplementary Table 13**. Correlation between 13 miRNAs co-expressed with hsa-let-7i and clinicopathological features

| **miRNA** | **Measurement** | **Ethnicity** | **Gender** | **Height** | **Race** | **Viral hepatitis infection** | **Vital status** | **Weight** |
| --- | --- | --- | --- | --- | --- | --- | --- | --- |
| hsa-miR-145 | Correlation Coefficient | -0.079 | 0.029 | 0.092 | .128* | .115* | -0.044 | .108* |
|  | Sig. (2-tailed) | 0.125 | 0.567 | 0.075 | 0.013 | 0.026 | 0.397 | 0.037 |
| hsa-miR-10a | Correlation Coefficient | -0.041 | -0.019 | 0.073 | .104* | 0.097 | 0.033 | 0.035 |
|  | Sig. (2-tailed) | 0.427 | 0.701 | 0.159 | 0.045 | 0.060 | 0.520 | 0.498 |
| hsa-let-7b | Correlation Coefficient | -0.064 | 0.051 | 0.014 | -0.052 | 0.085 | -0.030 | -0.067 |
|  | Sig. (2-tailed) | 0.211 | 0.324 | 0.775 | 0.313 | 0.100 | 0.558 | 0.193 |
| hsa-miR-155 | Correlation Coefficient | -0.018 | 0.057 | 0.034 | 0.019 | 0.028 | -.145** | .153** |
|  | Sig. (2-tailed) | 0.728 | 0.265 | 0.511 | 0.702 | 0.590 | 0.005 | 0.003 |
| hsa-miR-142 | Correlation Coefficient | 0.007 | 0.048 | 0.023 | 0.021 | 0.018 | -.113* | .124* |
|  | Sig. (2-tailed) | 0.889 | 0.350 | 0.650 | 0.679 | 0.722 | 0.028 | 0.017 |
| hsa-miR-125a | Correlation Coefficient | -0.07431 | -0.054 | 0.010 | -0.024 | .130* | -0.037 | -0.097 |
|  | Sig. (2-tailed) | 0.152 | 0.293 | 0.835 | 0.632 | 0.011 | 0.476 | 0.061 |
| hsa-miR-199a-1 | Correlation Coefficient | -0.079 | 0.016 | 0.058 | 0.016 | 0.070 | -0.036 | 0.019 |
|  | Sig. (2-tailed) | 0.125 | 0.745 | 0.260 | 0.757 | 0.177 | 0.477 | 0.701 |
| hsa-miR-214 | Correlation Coefficient | -0.073 | 0.007 | 0.073 | 0.053 | 0.071 | -0.034 | 0.050 |
|  | Sig. (2-tailed) | 0.155 | 0.887 | 0.158 | 0.306 | 0.166 | 0.507 | 0.331 |
| hsa-miR-424 | Correlation Coefficient | -0.045 | -0.019 | 0.050 | 0.067 | 0.084 | 0.053 | 0.076 |
|  | Sig. (2-tailed) | 0.380 | 0.705 | 0.328 | 0.194 | 0.105 | 0.304 | 0.142 |
| hsa-miR-708 | Correlation Coefficient | -0.047 | -0.0001 | 0.068 | .113* | 0.089 | -0.090 | 0.067 |
|  | Sig. (2-tailed) | 0.363 | 0.998 | 0.186 | 0.028 | 0.083 | 0.082 | 0.194 |
| hsa-miR-542 | Correlation Coefficient | -0.080 | 0.006 | 0.043 | -0.033 | 0.064 | -0.013 | 0.018 |
|  | Sig. (2-tailed) | 0.120 | 0.901 | 0.403 | 0.521 | 0.216 | 0.795 | 0.729 |
| hsa-miR-342 | Correlation Coefficient | -0.047 | 0.044 | 0.045 | 0.045 | 0.021 | -0.082 | .138** |
|  | Sig. (2-tailed) | 0.361 | 0.388 | 0.379 | 0.383 | 0.682 | 0.111 | 0.007 |
| hsa-miR-450a-1 | Correlation Coefficient | -0.071 | 0.012 | .122* | 0.075 | 0.096 | 0.002 | .102* |
|  | Sig. (2-tailed) | 0.170 | 0.813 | 0.0185 | 0.1457 | 0.062 | 0.956 | 0.048 |

**. Correlation is significant at the 0.01 level (2-tailed).

*. Correlation is significant at the 0.05 level (2-tailed).

# References

1. Tian Q, Liang L, Ding J, Zha R, Shi H, Wang Q, et al. MicroRNA-550a Acts as a Pro-Metastatic Gene and Directly Targets Cytoplasmic Polyadenylation Element-Binding Protein 4 in Hepatocellular Carcinoma. PLOS ONE 2012;7(11):e48958 doi 10.1371/journal.pone.0048958.
2. Yang JZ, Bian L, Hou JG, Wang HY. MiR-550a-3p promotes non-small cell lung cancer cell proliferation and metastasis through down-regulating TIMP2. European review for medical and pharmacological sciences 2018;22(13):4156-65 doi 10.26355/eurrev_201807_15408.
3. Ho J-Y, Hsu R-J, Wu C-H, Liao G-S, Gao H-W, Wang T-H, et al. Reduced miR-550a-3p leads to breast cancer initiation, growth, and metastasis by increasing levels of ERK1 and 2. Oncotarget 2016;7(33):53853-68 doi 10.18632/oncotarget.10793.
4. Balaguer F, Moreira L, Lozano JJ, Link A, Ramirez G, Shen Y*, et al.* Colorectal cancers with microsatellite instability display unique miRNA profiles. Clinical cancer research : an official journal of the American Association for Cancer Research **2011**;17(19):6239-49 doi 10.1158/1078-0432.ccr-11-1424.
5. Lee CH, Kuo WH, Lin CC, Oyang YJ, Huang HC, Juan HF. MicroRNA-regulated protein-protein interaction networks and their functions in breast cancer. International journal of molecular sciences **2013**;14(6):11560-606 doi 10.3390/ijms140611560. 24
6. Zheng J, Dong P, Gao S, Wang N, Yu F. High expression of serum miR-17-5p associated with poor prognosis in patients with hepatocellular carcinoma. Hepato-gastroenterology **2013**;60(123):549-52 doi 10.5754/hge12754.
7. Wang W, Zhao LJ, Tan YX, Ren H, Qi ZT. Identification of deregulated miRNAs and their targets in hepatitis B virus-associated hepatocellular carcinoma. World journal of gastroenterology **2012**;18(38):5442-53 doi 10.3748/wjg.v18.i38.5442. 22
8. Zheng J, Sadot E, Vigidal JA, Klimstra DS, Balachandran VP, Kingham TP*, et al.* Characterization of hepatocellular adenoma and carcinoma using microRNA profiling and targeted gene sequencing. PLoS One **2018**;13(7):e0200776 doi 10.1371/journal.pone.0200776.
9. Augello C, Gianelli U, Savi F, Moro A, Bonoldi E, Gambacorta M*, et al.* MicroRNA as potential biomarker in HCV-associated diffuse large B-cell lymphoma. Journal of clinical pathology **2014**;67(8):697-701 doi 10.1136/jclinpath-2014-202352.
10. Shih TC, Tien YJ, Wen CJ, Yeh TS, Yu MC, Huang CH*, et al.* MicroRNA-214 downregulation contributes to tumor angiogenesis by inducing secretion of the hepatoma-derived growth factor in human hepatoma. Journal of hepatology **2012**;57(3):584-91 doi 10.1016/j.jhep.2012.04.031.
11. Lin M, Chen W, Huang J, Gao H, Ye Y, Song Z*, et al.* MicroRNA expression profiles in human colorectal cancers with liver metastases. Oncology reports **2011**;25(3):739-47 doi 10.3892/or.2010.1112.
12. Jiang L, Wang Y, Rong Y, Xu L, Chu Y, Zhang Y*, et al.* miR-1179 promotes cell invasion through SLIT2/ROBO1 axis in esophageal squamous cell carcinoma. International journal of clinical and experimental pathology **2015**;8(1):319-27.
13. Hu B, Tang WG, Fan J, Xu Y, Sun HX. Differentially expressed miRNAs in hepatocellular carcinoma cells under hypoxic conditions are associated with transcription and phosphorylation. Oncology letters **2018**;15(1):467-74 doi 10.3892/ol.2017.7349.
14. Yu L, Ding GF, He C, Sun L, Jiang Y, Zhu L. MicroRNA-424 is down-regulated in hepatocellular carcinoma and suppresses cell migration and invasion through c-Myb. PLoS One **2014**;9(3):e91661 doi 10.1371/journal.pone.0091661.
15. Morishita A, Iwama H, Fujihara S, Sakamoto T, Fujita K, Tani J*, et al.* MicroRNA profiles in various hepatocellular carcinoma cell lines. Oncology letters **2016**;12(3):1687-92 doi 10.3892/ol.2016.4853.
16. Ma J, Li Y, Yao L, Li X. Analysis of MicroRNA Expression Profiling Involved in MC-LR-Induced Cytotoxicity by High-Throughput Sequencing. Toxins **2017**;9(1):23 doi 10.3390/toxins9010023.
17. Zhao N, Sun B-C, Zhao X-L, Wang Y, Meng J, Che N*, et al.* Role of Bcl-2 and its associated miRNAs in vasculogenic mimicry of hepatocellular carcinoma. International journal of clinical and experimental pathology **2015**;8(12):15759-68.
18. Wu L, Wang Q, Yao J, Jiang H, Xiao C, Wu F. MicroRNA let-7g and let-7i inhibit hepatoma cell growth concurrently via downregulation of the anti-apoptotic protein B-cell lymphoma-extra large. Oncology letters **2015**;9(1):213-8 doi 10.3892/ol.2014.2706.
19. Lu C, Liao Z, Cai M, Zhang G. MicroRNA-320a downregulation mediates human liver cancer cell proliferation through the Wnt/β-catenin signaling pathway. Oncology letters **2017**;13(2):573-8 doi 10.3892/ol.2016.5479.
20. Xie F, Yuan Y, Xie L, Ran P, Xiang X, Huang Q*, et al.* miRNA-320a inhibits tumor proliferation and invasion by targeting c-Myc in human hepatocellular carcinoma. OncoTargets and therapy **2017**;10:885-94 doi 10.2147/ott.s122992.
